# The health burden and racial-ethnic disparities of air pollution from the major oil and gas lifecycle stages in the United States

**DOI:** 10.1126/sciadv.adu2241

**Published:** 2025-08-22

**Authors:** Karn Vohra, Eloise A. Marais, Ploy Achakulwisut, Susan Anenberg, Colin Harkins

**Affiliations:** ^1^ Department of Geography, University College London, London, UK.; ^2^ Stockholm Environment Institute, Seattle, WA, USA.; ^3^ Milken Institute School of Public Health, George Washington University, Washington, DC, USA.; ^4^ Cooperative Institute for Research in Environmental Sciences, University of Colorado Boulder, Boulder, CO, USA.; ^5^ NOAA Chemical Sciences Laboratory, Boulder, CO, USA.

## Abstract

The United States has one of the world’s largest oil and gas (O&G) industries, yet the health impacts and inequities from pollutants produced along the O&G lifecycle remain poorly characterized. Here, we model the contribution of major lifecycle stages (upstream, midstream, downstream, and end-use) to air pollution and estimate the associated chronic health outcomes and racial-ethnic disparities across the contiguous US in 2017. We estimate lifecycle annual burdens of 91,000 premature deaths attributable to fine particles (PM_2.5_), nitrogen dioxide (NO_2_), and ozone, 10,350 PM_2.5_-attributable preterm births, 216,000 incidences of NO_2_-attributable childhood-onset asthma, and 1610 lifetime cancers attributable to hazardous air pollutants (HAPs). Racial-ethnic minorities experience the greatest disparities in exposure and health burdens across almost all lifecycle stages. The greatest absolute disparities occur for Black and Asian populations from PM_2.5_ and ozone, and the Asian population from NO_2_ and HAPs. Relative inequities are most extreme from downstream activities, especially in Louisiana and Texas.

## Teaser

Largest total population health burden is from O&G end-use, but greatest relative disparities are linked to downstream activities.

## INTRODUCTION

The United States (US) is the world’s largest oil and natural gas (O&G) producer (*1*). The O&G industry accounts for 8% of the US economy (*2*). Since around 2008, substantial expansion of O&G exploration and extraction and increasing consumption of O&G-derived products have been spurred by a combination of technological progress enabling the widespread production of unconventional O&G, cheaper O&G products, and decline in demand for coal (*3*, *4*). Yet, there is growing recognition of the need to phase out O&G to meet the Paris Agreement’s temperature goal (*5*–*7*), as well as increasing concerns among the public, researchers, and policymakers over the local health impacts of air, water, and waste pollution from O&G activities in the US, particularly the disproportionate burden on marginalized groups (*8*–*11*).

The O&G lifecycle includes four major stages: “upstream” exploration and extraction; “midstream” storage and transmission; “downstream” alterations to the extracted material through processes like oil refining, gas processing, and synthesis of petrochemical products; and “end-use” activities that include additional storage and transmission to reach consumers and ultimate consumption of O&G products for energy and non-energy purposes. All these stages produce air pollutants that either directly harm public health or undergo reactions in the atmosphere to form additional health-damaging pollutants (*12*). Activities such as well drilling and completion, hydraulic fracturing, venting and flaring, oil refining, gas processing, and O&G combustion in the transportation and power sectors are all direct or precursor sources of fine particulate matter (PM_2.5_), nitrogen dioxide (NO_2_), and ozone (O_3_) (*13*–*19*). The O&G sector is the largest industrial source of volatile organic compounds (VOCs) in the US (*20*). O&G production also generates hazardous air pollutants (HAPs) and radioactive by-products, enhancing airborne radioactivity downwind of extraction sites (*21*, *22*). The health outcomes of concern from long-term exposure to air pollutants from the O&G lifecycle include, but are not limited to, multi-cause premature deaths (*23*–*25*) and preterm births (*26*) for PM_2.5_, premature death from chronic respiratory diseases for O_3_ (*27*), childhood-onset asthma for NO_2_ (*28*), and cancers for HAPs (*29*). There are many more adverse health outcomes that have been attributed to specific O&G lifecycle stages that are more challenging to quantify, due to lack of robust information relating exposure to health risk. For upstream and midstream activities, these include migraines, fatigue, depression, high-risk pregnancy, gestational hypertension and eclampsia, sleeping disorders, and endocrine and developmental disruption such as neural tube and oral cleft defects (*30*–*38*). Downstream activities include low birth weight, spontaneous abortion, congenital malformations, and diseases of the kidney, liver, and thyroid (*39*). End-use activities include adverse reproductive outcomes and respiratory illnesses such as wheezing (*40*). It is also increasingly apparent that health risks linked to exposure to air pollutants from the O&G sector are most severe for marginalized ethnic communities in the US (*9*, *10*, *41*–*44*). Unequal exposure to air pollution has been identified for all O&G lifecycle stages, due to discriminatory siting of infrastructures such as upstream extraction wells, midstream pipelines and compressor stations (*10*, *11*), and downstream chemical manufacturing plants (*11*, *45*), and also from greater flaring intensity of natural gas close to marginalized ethnic communities (*10*). Disproportionate exposure to end-use sources like vehicle traffic results from historical redlining and related practices (*46*, *47*) that place marginalized communities closer to busy, dense road networks (*48*). Key among other influential factors synthesized and discussed by Banzhaf *et al.* (*49*) is community bargaining power that determines effective environmental regulation. Given this growing evidence, there is an urgent need for targeted studies that identify and quantify injustices in exposure to inform actions that alleviate the most burdened communities (*9*, *49*).

Past studies that have quantified total population health burdens attributable to exposure to outdoor air pollution from the O&G sector in the US have limited focus to a single lifecycle stage (*50*, *51*), a single pollutant (predominantly PM_2.5_) (*52*), or specific regions like the Appalachian natural gas basin (*53*). Others have lumped O&G with coal to determine the overall health burden of fossil fuel combustion (*54*, *55*) or used reduced complexity chemistry models (*56*) that do not suitably represent chemical reactions forming secondary O_3_ and PM_2.5_ or the influence of O&G emissions on the oxidative capacity of the atmosphere affecting atmospheric abundances of carcinogenic volatiles like formaldehyde with large secondary sources (*57*, *58*). Studies examining environmental injustices have progressed substantially since the 2003 review of the global oil industry by O’Rourke and Connolly (*11*). Still, these are so far limited to specific aspects of the US O&G lifecycle, such as the natural gas lifecycle only (*10*), national unconventional O&G wells (*59*, *60*), and upstream production wells in California (*42*). All have focused on air pollution exposure inequities (*42*, *59*), but not disparities in the resulting health burden that may be exacerbated by differences in underlying disease rates (*61*). A consistent quantification of the adverse health outcomes and inequities associated with the myriad air pollutants from individual stages of the entire US O&G lifecycle is crucial for policymakers to assess the benefits of transitioning to less polluting alternatives and identify the most adversely affected population subgroups to prioritize to address exposure and health inequities.

Here, we assess the influence of the full lifecycle of O&G activities on air pollution exposure and health burden, and the disparities in these, across the contiguous US (CONUS). We achieve this by first constructing a unified emissions inventory of each of the four main stages of the O&G lifecycle. This is then fed to the chemical transport model GEOS-Chem, simulated at high spatial resolution over the CONUS. Modeled ambient surface concentrations of health-harming air pollutants are applied to epidemiologically informed health risk assessment models to quantify the total premature mortality and incidences of preterm birth, asthma, and cancer attributable to exposure to O&G-derived pollutants. Air pollution exposures and attributable health burdens are then used with demographic datasets to examine racial and ethnic disparities in exposures and health outcomes.

## RESULTS

### Influence of emissions from O&G activities on ambient air pollution

**Fig. 1.**
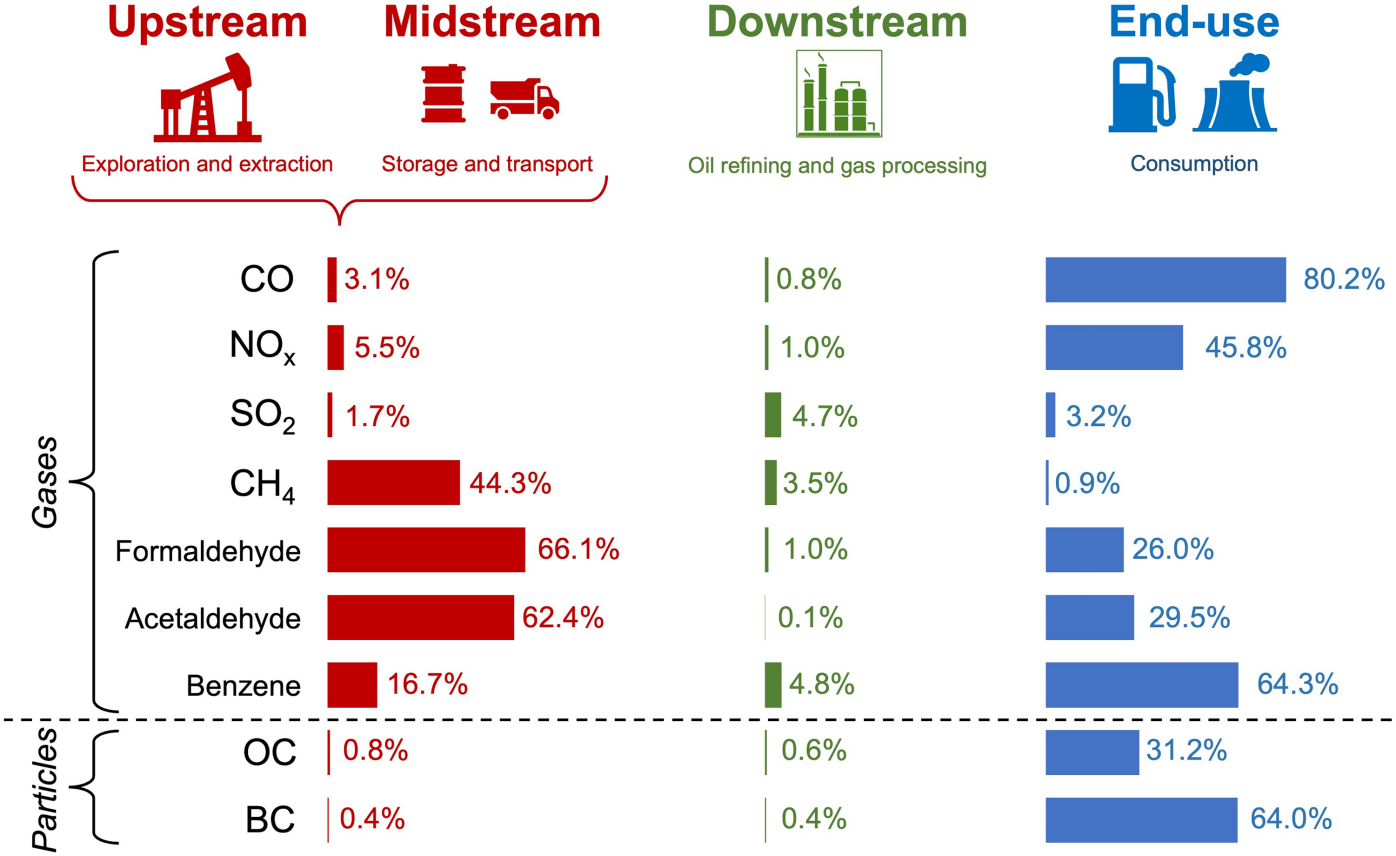
Contribution of air pollutant precursor emissions from major O&G lifecycle stages to total anthropogenic emissions for the CONUS in 2017. Shown are combined upstream and midstream (red), downstream (green), and end-use (blue) stages. Values outside the bars are the percent contribution of each stage to total anthropogenic emissions of 30 Tg of CO, 11 Tg of NO*_x_*, 5 Tg of SO_2_, 27 Tg of CH_4_, 0.3 Tg of formaldehyde, 0.2 Tg of acetaldehyde, 0.1 Tg of benzene, 0.7 Tg of OC, and 0.2 Tg of BC. Dotted line separates gas- and particle-phase precursors.

The unified inventory we construct is for the year 2017, as this is the most recent year of reported emissions across all datasets used. The steps and datasets to construct this inventory are detailed in the “Hourly, gridded air pollutant emissions of individual O&G lifecycle stages” section. Figure 1 shows the percent contribution of select precursor emissions of air pollutants from major lifecycle stages to total anthropogenic emissions in the US. The compounds in Fig. 1 include the HAPs formaldehyde, acetaldehyde, and benzene, nitrogen oxides (NO*_x_*), carbon monoxide (CO), methane (CH_4_), sulfur dioxide (SO_2_), organic carbon (OC), and black carbon (BC). HAPs and the NO*_x_* constituent NO_2_ are directly harmful to health. VOCs, including the HAPs in Fig. 1, NO*_x_*, and CO react to form O_3_ pollution, BC and OC are dominant (~80%) primary sources of PM_2.5_, and select VOCs, NO*_x_*, and SO_2_ are secondary sources of PM_2.5_ pollution forming secondary organic aerosols for VOCs, aerosol nitrate for NO*_x_*, and aerosol sulfate for SO_2_. Other carcinogenic HAPs like 1,3-butadiene formed from end-use activities such as combustion engines are absent, as these are not in the GEOS-Chem model version we use and have only been recently added. We do include other noncarcinogenic HAPs such as xylene and toluene in the inventory, but the contribution of emissions of these from O&G activities to ambient annual concentrations is orders of magnitude below reference concentrations associated with adverse health outcomes (0.1 mg m^−3^ for xylene, 5 mg m^−3^ for toluene; https://iris.epa.gov/AtoZ/; last accessed 10 February 2025). Emissions from upstream and midstream activities are combined, as we find that midstream activities alone are a negligible component (<1%) of total anthropogenic emissions for all pollutants except for VOCs. For individual VOCs, the contribution of midstream activities to total anthropogenic emissions ranges from 5 to 32%.

The O&G lifecycle accounts for more than half of CO, NO*_x_*, formaldehyde, acetaldehyde, benzene, and BC anthropogenic emissions, dominated by end-use activities for CO, NO*_x_*, benzene, and BC, and by midstream activities for formaldehyde and acetaldehyde. The largest O&G sources of these pollutants are end-use mobile sources for CO (*62*, *63*), NO*_x_* (*64*), and benzene (*65*), diesel vehicles (a subset of mobile sources) for BC (*64*, *66*), and midstream compressor stations for formaldehyde and acetaldehyde (*67*). Benzene from mobile sources results from vehicle exhaust and evaporative emissions of unburned fuel (*65*), while formaldehyde and acetaldehyde from midstream compressor stations result from the inefficient combustion of exhaust gas that provides the energy needed for pipeline transport of natural gas (*68*, *69*). Large upstream and midstream emissions of CH_4_, a greenhouse gas and surface O_3_ precursor, are due to leaking, venting, and inefficient flaring (*70*).

**Fig. 2.**
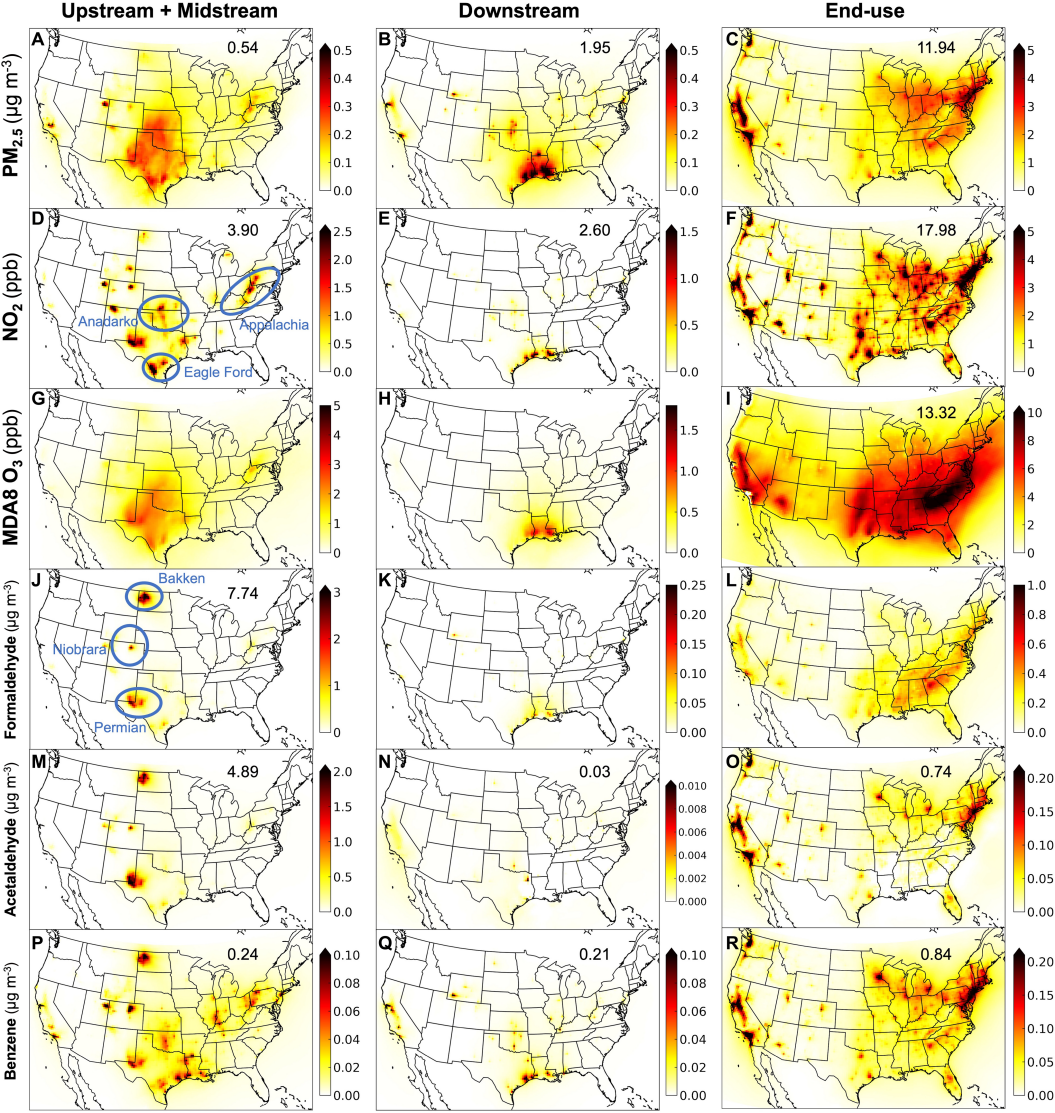
Surface concentrations of health-damaging air pollutants for each major O&G lifecycle stage in 2017. These are peak-season means for MDA8 O_3_ (**G** to **I**) and annual means for all other pollutants (**A** to **F** and **J** to **R**). Inset values are maximum values where the color bar saturates. Note that different color scales are used.

Figure 2 shows the surface concentrations of health-harming air pollutants resulting from precursor emissions in each of the major O&G lifecycle stages (Fig. 1), as simulated by GEOS-Chem (“Air pollutant modeling and validation” section). The annual mean PM_2.5_ concentration from the full O&G lifecycle averaged over the CONUS is 1.2 μg m^−3^. This is mostly (~80%) secondary PM_2.5_ and accounts for almost one-fifth (17%) of the US annual mean PM_2.5_ concentration resulting from all emissions sources. End-use activities lead to the largest enhancements in all pollutants, except formaldehyde and acetaldehyde that are most enhanced from combined upstream and midstream activities. End-use annual mean PM_2.5_ averages ~3 to 4 μg m^−3^ in the northeastern US states of New Jersey and Connecticut, and in the District of Columbia. End-use annual mean NO_2_, mainly from traffic, exceeds 10 parts per billion (ppb) in major cities along the northeast corridor (Fig. 2F). The southeast US includes a coincident band of enhanced end-use formaldehyde of up to 1 μg m^−3^ (Fig. 2L) and of >10 ppb maximum daily 8-hour running-mean ozone (MDA8 O_3_) (Fig. 2I), the metric used to assess health risk of exposure to O_3_. The southeast US band of enhanced MDA8 O_3_ is because NO*_x_* from end-use activities increases the yields of formaldehyde and other reactive VOCs from biogenic isoprene oxidation in a region where O_3_ formation is limited by the availability of VOCs (*58*). End-use benzene and acetaldehyde are mostly from unburned fuel exhaust emissions, released as primary emissions for benzene and also produced from oxidation of alkanes and alkenes for acetaldehyde.

The influence of combined upstream and midstream stages on ambient air pollution is most pronounced in the O&G-producing Permian and Eagle Ford basins in Texas, the Anadarko in Oklahoma, the Bakken in North Dakota, the Niobrara in Colorado, and, to a lesser extent, the northeast portion of the Appalachian basin. PM_2.5_ from combined upstream and midstream stages averages ~0.2 μg m^−3^ in Texas, Oklahoma, and Kansas (Fig. 2A). Secondary PM_2.5_ is from emissions of NO*_x_* from drill rigs and pump jacks in the O&G basins in Texas that cause NO*_x_* enhancements of 2 to 4 ppb (Fig. 2D). This NO*_x_*, in turn, influences peak-season combined upstream and midstream MDA8 O_3_ that averages ~2 ppb in Texas, Oklahoma, and Kansas (Fig. 2G). Combined upstream and midstream activities are a large source of fugitive emissions of CH_4_ that has the potential to contribute to O_3_ pollution (*71*). We conduct an additional separate sensitivity simulation without CH_4_ emissions to find that CH_4_ emissions alone are only a small (<0.1 ppb) contributor to O_3_ in comparison to the enhancements plotted in Fig. 2 (G to I). This may be because of the resolution of the model or because the inventory does not include time-variant, intermittent, but substantial super emissions of CH_4_ (*72*). The large concentrations of combined upstream and midstream formaldehyde and acetaldehyde of >2 μg m^−3^ for each pollutant in the Bakken, Niobrara, and Permian basins (Fig. 2, J and M) are from compressor station emissions (*67*).

Most downstream air pollution manifests as concentrated, isolated enhancements at locations with large oil-refining activities in eastern Texas and in southern Louisiana. Air pollution from oil-refining activity near San Francisco and Los Angeles in California is only apparent for PM_2.5_ and NO_2_ with peak enhancements of 1.1 μg m^−3^ for PM_2.5_ (Fig. 2B) and 0.6 ppb for NO_2_ (Fig. 2E). Contributions of downstream activities in California to other air pollutants are minor at <0.1 ppb for MDA8 O_3_ and <0.2 μg m^−3^ for HAPs.

The contribution of individual O&G lifecycle stages to surface concentrations of the carcinogen benzene (Fig. 2, P to R) is less than formaldehyde for all stages, comparable to end-use acetaldehyde for end-use, and only exceeds acetaldehyde for the downstream stage. Benzene emissions are 52 to 64% less than emissions of formaldehyde and acetaldehyde that also include secondary formation from oxidation of VOC precursors.

### Public health burden of ambient air pollution from the O&G lifecycle

**Fig. 3.**
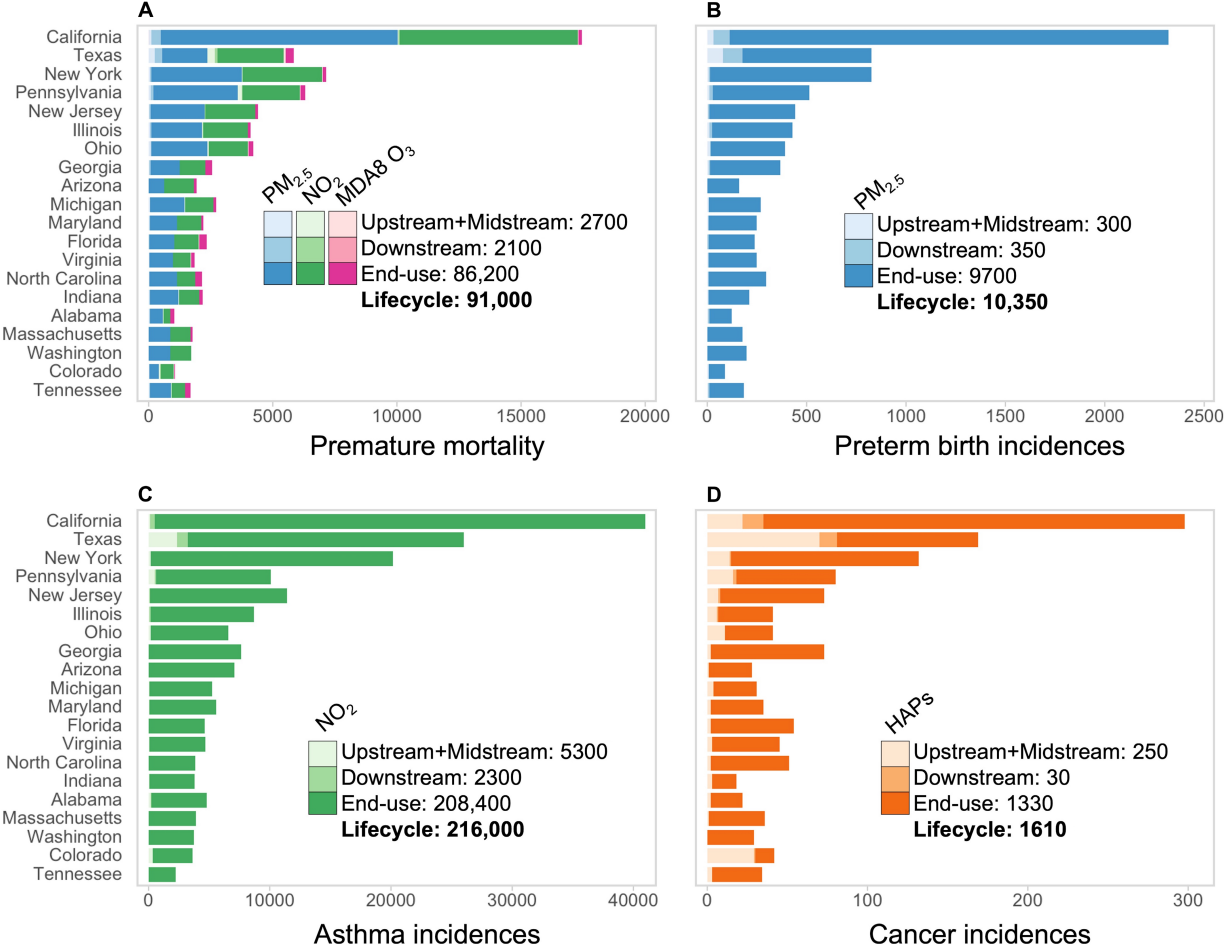
Health burden of ambient air pollution linked to major O&G lifecycle stages for the 20 most affected US states. Outcomes are PM_2.5_-attributable adult (blues), NO_2_-attributable elderly (greens), and MDA8 O_3_–attributable all-age (pinks) premature mortality (**A**), PM_2.5_-attributable preterm birth incidences (blues) (**B**), NO_2_-attributable childhood asthma incidences (greens) (**C**), and HAP-attributable lifetime cancer incidences (oranges) (**D**). Color intensity denotes each O&G lifecycle stage. Bars rank states by most to least total health burden. Values inset are US total health burdens for each lifecycle stage and the total (in bold). Data for all 48 CONUS states and the District of Columbia (DC) are in tables S2 to S7.

Figure 3 ranks the 20 CONUS states with the greatest total health burden from the O&G lifecycle, estimated using the modeled air pollutant concentrations in Fig. 2, epidemiologically informed health risk assessment models, population demographics data, and baseline rates of mortality and incidences of preterm births and asthma (“Quantification of adverse health outcomes” section). The health burdens of air pollution from the O&G lifecycle are 47,200 [uncertainty interval (UI): 41,600 to 53,100] for PM_2.5_-attributable non-accidental adult (25+ years) premature deaths, 39,100 (UI: 36,700 to 42,500) for NO_2_-attributable non-accidental elderly (65+ years) premature deaths, 4700 (UI: 1200 to 8100) for MDA8 O_3_–attributable chronic obstructive pulmonary disease (COPD) premature deaths across all population age groups, 10,350 (UI: 3500 to 17,500) for PM_2.5_-attributable preterm birth incidences, 216,000 (UI: 42,000 to 316,300) for NO_2_-attributable onset asthma for children aged 1 to 18 years, and 1610 for HAP-attributable lifetime cancers. The estimated HAP-attributable cancer incidences equate to 21 cancer occurrences in 2017 for a US-average statistical lifespan of 76.4 years. A complete phaseout of O&G and its associated air pollution in the US would decrease PM_2.5_ exposure for 13 million people to below 2.5 μg m^−3^, the annual mean risk threshold of premature mortality (“Quantification of adverse health outcomes” section). Similarly, an additional 1.2 million people would live below the MDA8 O_3_ risk threshold of 32.4 ppb and 148 million people below the NO_2_ risk threshold of 2 ppb.

Texas and California have the greatest burden for almost all pollutant-health outcome risks and O&G lifecycle stages. Only New York (7200) and Pennsylvania (6300) premature mortality totals for all stages from combined exposure to PM_2.5_, NO_2_, and MDA8 O_3_ surpass that for Texas (5800). The ranking in Fig. 3 aligns closely with state total populations. If the health incidences are population-normalized, the top 5 ranked states are instead New Jersey, the District of Columbia, New York, California, and Maryland. The 20 states shown in Fig. 3 account for 84% of the CONUS total incidences across all risks. More than half (53%) of the total CONUS health burden is in the six top-ranked states in Fig. 3 (California, Texas, New York, Pennsylvania, New Jersey, and Illinois) that account for about one-third of the CONUS population. End-use activity air pollution has the greatest public health burden, accounting for 96% of total incidences for all CONUS states and for the states in Fig. 3. Non–end-use activities make an appreciable contribution to chronic risk incidences for premature mortality, asthma, and preterm births in Texas and California, and for cancer incidences in states with large upstream activities: Texas, California, New York, Pennsylvania, Ohio, and Colorado (Fig. 2, J, M, and P).

End-use activities dominate all incidences, except for cancer risk in specific states with large O&G extraction basins, such as Colorado, Texas, and North Dakota (fig. S1). Across CONUS, the end-use stage accounts for ~94% of O&G lifecycle PM_2.5_-attributable deaths, ~96% of NO_2_-attributable deaths, ~90% MDA8 O_3_–attributable COPD deaths, ~94% of PM_2.5_-attributable preterm birth incidences, ~91% NO_2_-attributable pediatric asthma incidences, and ~82% HAP-attributable cancer incidences. Of the HAPs, formaldehyde accounts for most (~68%) of the burden, followed by benzene (~26%) and acetaldehyde (~6%). The larger health burden attributable to benzene than acetaldehyde, despite the relatively small surface concentrations of benzene (Fig. 2), is because benzene has a 3.5 times higher cancer risk than acetaldehyde (*29*, *73*, *74*).

Greater burden of end-use activities for MDA8 O_3_ is because downstream activity emissions of the O_3_ precursors NO*_x_* and VOCs are considerably less than the other activities (Fig. 1), resulting in at most <1 ppb downstream MDA8 O_3_ (Fig. 2K) compared to >10 ppb end-use MDA8 O_3_ (Fig. 2I).

US states that neighbor those with intensive upstream, midstream, or downstream activities are also affected by transported pollution, as PM_2.5_ can persist in the atmosphere for a few days. Hotspots of shorter-lived NO_2_ in Fig. 2 (D and E) help to identify the location of activities with large emissions intensities of PM_2.5_ precursors that are displaced from the source, leading to the widespread enhancements in PM_2.5_ concentrations in Fig. 2 (A and B). For example, this PM_2.5_ enhancement is evident over Kansas, a state with far less local primary or precursor PM_2.5_ emissions from upstream activities than Oklahoma and Texas. The 30 upstream-activity premature deaths in Kansas can be linked to PM_2.5_ resulting from upstream activities in the Anadarko basin in Oklahoma and the Permian basin in Texas. Similarly, the 20 premature deaths in Arkansas can be linked to PM_2.5_ from downstream activities originating in Louisiana and Texas, as local emissions in Arkansas are less than emissions in these states. US O&G emissions also affect air quality in neighboring countries. As our model domain also includes southern Canada and northern Mexico (Fig. 2, “Air pollutant modeling and validation” section), we quantify the influence of US O&G activities on these neighboring countries. Population-weighted PM_2.5_ from all US O&G lifecycle stages is 0.8 μg m^−3^ in southern Canada and 0.5 μg m^−3^ in northern Mexico and is associated with 1170 premature deaths in southern Canada and 440 in northern Mexico.

To put the PM_2.5_-, NO_2_-, and MDA8 O_3_–attributable premature deaths, PM_2.5_-attributable preterm births, and NO_2_-attributable pediatric asthma incidences determined for the O&G lifecycle into context, we also compare the estimates in Fig. 3 to the total health burden due to all air pollutant sources (“Air pollutant modeling and validation” section). The O&G lifecycle accounts for ~20% of adult premature mortality linked to long-term exposure to PM_2.5_, 42% for COPD-related premature deaths from exposure to peak-season MDA8 O_3_, 24% of PM_2.5_-attributable preterm birth incidences, 22% of HAP-attributable cancer incidences, and 86% of elderly mortality and pediatric asthma incidences linked to exposure to NO_2_. Compared to the total health burden from all risks, the O&G lifecycle air pollution health burden is 2% of all non-accidental mortality in adults and in the elderly population, 3% of COPD mortality for all age groups, 3% of preterm births, 10% of pediatric asthma incidences, and 4% of all respiratory and hematologic cancers in the US (tables S2 to S7).

### Racial and ethnic disparities in air pollution and attributable health burden linked to the O&G lifecycle

**Fig. 4.**
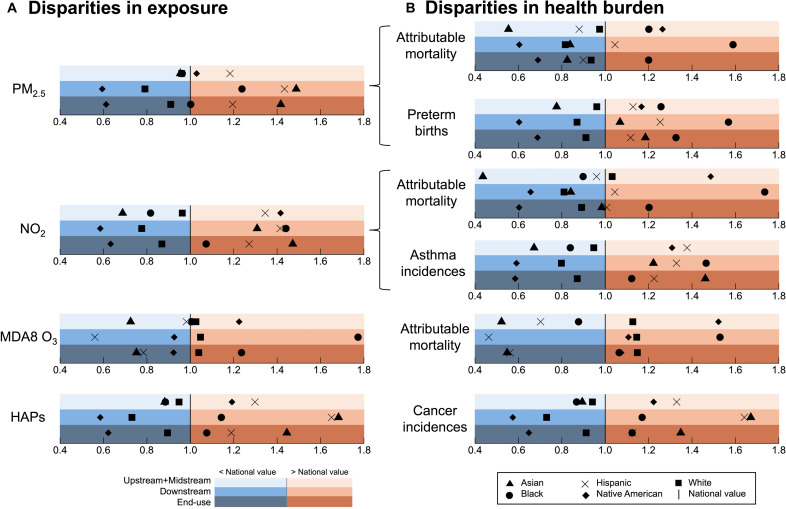
US racial and ethnic disparities in air pollution exposure and attributable health burden linked to major O&G lifecycle stages. Disparities are relative to the national value. Disparities are estimated as population-weighted mean concentrations for exposure (**A**) and using age-standardized baseline rates of mortality or incidences for health burdens (**B**) (“Exposure and health burden disparity analyses” section). Background colors separate disparities above (reds) and below (blues) the national value. Color intensity denotes each O&G lifecycle stage. Symbols discern population subgroups: Hispanic (cross) and non-Hispanic Asian (triangle), Black (circle), Native American (diamond), and white (square). Relative disparity of 0.26 in MDA8 O_3_ exposure for the Asian population is outside the range plotted.

We estimate disparities by comparison of exposures and population-standardized health burdens for each racial and ethnic group to the national average for the total population, as detailed in the “Exposure and health burden disparity analyses” section. Figure 4 shows the relative disparities estimated by normalizing to the national value, enabling comparisons across different O&G lifecycle stages, pollutants, health outcomes, and population totals. Absolute racial and ethnic disparities for the end-use stage are 3 to 101 times more than the other stages (table S8), because of the much greater contribution of end-use activities to air pollution and attributable burdens (Figs. 2 and 3). Asian, Black, Hispanic, and Native American groups experience the worst exposures and burdens for all lifecycle stages and pollutants, except end-use MDA8 O_3_–attributable mortality. The greatest relative disparities almost all occur in the downstream stage, a lifecycle stage that is no more than 3% of the health endpoint incidences for the total population (Fig. 3). The only instance where downstream does not account for the greatest relative disparity is NO_2_ exposure. Relative disparities for NO_2_ asthma burden are equal worst for end-use and downstream stages.

For the downstream activities, the Black population experiences the greatest burden for PM_2.5_-, NO_2_-, and MDA8 O_3_–attributable mortality, PM_2.5_-attributable preterm births, and NO_2_-attributable asthma incidences. This is because of coincidence of southern Louisiana and eastern Texas pollutant hotspots of downstream PM_2.5_, NO_2_, and O_3_ (Fig. 2, B, E, and H), and relatively large age-standardized baseline mortality rates of >1600 per 100,000 people (fig. S3H) in locations where the Black population accounts for more than half the total population (fig. S3G). Downstream HAP-attributable exposures and cancers are worst for the Asian population, due to collocation of downstream benzene hotspots (Fig. 2Q) and high Asian population density in California and eastern Texas.

For the combined upstream and midstream stages, the Native American population experiences the most severe exposure to MDA8 O_**3**_ and NO_2_ and mortality burdens from PM_2.5_, MDA8 O_**3**_, and NO_2_ and the Hispanic population experiences the most severe exposure to PM_2.5_, and HAPs and health burden of asthma from NO_2_ and cancers from HAPs. End-use disparities are mixed in that the Asian population has the most severe exposures to PM_2.5_, NO_2_, and HAPs and burdens of asthma from NO_2_ and cancers from HAPs due to high Asian population densities in large urban areas. The Black population has the most severe exposure to MDA8 O_3_, mortality burden of PM_2.5_ and NO_2_ and preterm birth burden of PM_2.5_, and the white population has the most severe mortality burden of MDA8 O_3_. The latter is due to slightly greater COPD baseline mortality rates among the white population (fig. S3O).

## DISCUSSION

According to our results, the O&G lifecycle contributes to over half of precursor emissions of most health-harming air pollutants assessed. In 2017, this air pollution was linked to 91,000 premature deaths, attributed mostly to PM_2.5_ (52%) and NO_2_ (43%), and incidences of 10,350 preterm births for PM_2.5_, 216,000 childhood-onset asthma for NO_2_, and 1610 cancers for HAPs. The study of Buonocore *et al.* (*50*) determined the health burden of NO_2_, PM_2.5_, and MDA8 O_3_ from O&G production for individual states in CONUS in 2016, so we compare these to our health burdens for the combined upstream and midstream stage. Our estimated PM_2.5_-attributable premature mortality burden is 38% less than their estimate of 2100, as they used a health risk assessment model that yields ~1.5 times higher risk of premature mortality than other models (*54*). Our estimated MDA8 O_3_–attributable premature mortality burdens are not comparable, as their estimate is determined for all causes and so uses a much larger all-cause baseline mortality rate than we use for COPD only. The most recent epidemiological evidence links premature mortality from MDA8 O_3_ exposure to only COPD rather than all causes (*27*). Our estimate of NO_2_-attributable pediatric asthma incidences uses the same Khreis *et al.* (*28*) relative risk function as is used by Buonocore *et al.* (*50*), but our individual state incidences are mostly four times more than Buonocore *et al.* (*50*). Our combined upstream and midstream NO*_x_* emissions (0.88 Tg) are similar to those used in Buonocore *et al.* (*50*) (1 Tg). The difference may be the ~2 ppb underestimate in NO_2_ in the model used by Buonocore *et al.* (*50*) in densely populated California and northeast US where the health burned of NO_2_ is most severe. A value of ~2 ppb is ~40% of the GEOS-Chem–simulated end-use NO_2_ in Fig. 2F.

Ours is the first study to quantify national racial and ethnic disparities in exposure and health burden specific to air pollution for each major O&G stage, making it challenging to compare our findings with previous studies. Such a comparison is further complicated by different approaches to disparity analyses in earlier studies: focusing on most or least white and Hispanic population (*61*), or quantifying air pollution contribution to racial-ethnic disparities (*75*, *76*). One study examined national disparities in exposure to PM_2.5_, O_3_, and NO_2_ in 2010, but from all emissions sources. Similar to our results, highest national mean exposures from that study were identified for the marginalized population subgroups, specifically, non-Hispanic Black population for PM_2.5_ and non-Hispanic Asian population for NO_2_ and O_3_. For the health burden, our findings of greatest disparities in preterm birth incidences for the Black population across all major O&G lifecycle stages (Fig. 4B) are consistent with previously found racial disparities in exposure to air pollution from all sources in California (*75*). Gestation periods in that work were found to be shorter for Black than white women (*75*). Although our results are at the national level, California accounts for 22% of the estimated PM_2.5_-attributable preterm births linked to the O&G lifecycle (Fig. 3B).

Typically, environmental justice analyses are conducted at census-tract levels (*61*, *77*, *78*). Most census tracts are at finer spatial resolution than the GEOS-Chem model (~28 km) we use. Two separate studies have investigated the influence of resolution on absolute and relative disparities, both using the Intervention Model for Air Pollution (InMAP). Qiu *et al.* (*79*) determined similar relative disparities using InMAP with county-level and census-tract–level demographic data, whereas Paolella *et al.* (*80*) varied the spatial resolution of InMAP to conclude that finer model resolutions yield greater absolute disparities. This suggests that the absolute disparities we estimate are conservative using GEOS-Chem, whereas the relative disparities should be insensitive to the GEOS-Chem model resolution.

Our health burden and disparity estimates are subject to uncertainties and limitations from our choice of emission inventories, treatment of chemical formation and loss processes in GEOS-Chem, model and demographic data spatial resolutions, and health risk assessment models. Recent estimates show that the US Environmental Protection Agency’s (EPA) National Emissions Inventory substantially underestimates emissions from the entire O&G lifecycle by not accounting for the rapid growth in unconventional O&G activities, from undercounting facilities, and because of missing large emissions such as those from flaring and venting (*81*–*84*). Simulated upstream HAP concentrations using these emissions and a coarse-resolution model are orders of magnitude smaller than field observations (*67*, *85*, *86*), and as we do not include other HAPs like 1,3-butadiene, the HAP-attributable cancer risk and absolute disparities we estimate are likely underestimated.

There is epidemiological evidence for differential vulnerabilities of racial and ethnic groups, for example, the Black population is at greater risk of premature mortality from exposure to PM_2.5_ than the non-Hispanic white population (*76*), but these risks are unquantified for many subgroups and health outcomes covered in this study. Our asthma incidence disparity analysis also does not account for racial-ethnic variability in baseline asthma incidences. There are racial disparities in asthma prevalence among children in the US (*87*–*89*), with Black and Hispanic children having higher incidences and an earlier onset of asthma than white children (*89*). The Global Burden of Disease (GBD) state-level asthma incidences that we use are not stratified by race or ethnicity (“Exposure and health burden disparity analyses” section), and the US Centers for Disease Control (CDC) only reports race-specific asthma prevalences rather than incidences and only for a subset of US states (https://ephtracking.cdc.gov/DataExplorer/; last accessed 17 February 2025).

Premature mortality estimates are sensitive to the choice of health risk assessment models (*90*), due to differences in risk values and curve shapes, particularly for PM_2.5_. If instead we use the global exposure mortality model [GEMM; Burnett *et al.* (*24*)] to calculate PM_2.5_-attributable premature mortality, we estimate a burden of 51,300 (UI: 31,300 to 70,800) that is only 4100 more and within the uncertainty range (41,600 to 53,100) of our estimate.

GEOS-Chem has a positive bias in annual mean surface PM_2.5_ concentrations of 21% in comparison to the observation network (fig. S2). If we account for this bias by decreasing PM_2.5_ concentrations uniformly by 2.8 μg m^−3^ (the Theil-Sen regression intercept), PM_2.5_-attributable mortality for all emission sources decreases by 22%, but mortality attributable to PM_2.5_ from the O&G lifecycle increases by 14% from 47,200 to 54,000. This increase is because the gradient of the concentration-response curve increases with decline in PM_2.5_ (*91*).

We compute premature mortality from long-term exposure to three pollutants, PM_2.5_, NO_2_, and MDA8 O_3_, separately, so there is the potential for triple counting, as the risk of premature mortality from COPD occurs for all three pollutants. This is likely of most concern for the combined upstream and midstream and the downstream stages, as PM_2.5_ (Fig. 2, A and B), NO_2_ (Fig. 2, D and E), and MDA8 O_3_ have very similar spatial distributions (Fig. 2, G and H). These stages only account for a small portion (~5%) of total premature mortality from risks of exposure to these pollutants. In the three states, Texas, Oklahoma, and Kansas, with collocation of enhancements in PM_2.5,_ NO_2_, and MDA8 O_3_ from upstream, midstream, and downstream stages, premature mortality is 670 from PM_2.5_ exposure, 540 from NO_2_ exposure, and 130 from MDA8O_3_ exposure, accounting for ~1.5% of all O&G lifecycle premature deaths. COPD mortality is also only a small (6%) portion of all non-accidental premature deaths used to quantify premature mortality from exposure to PM_2.5_ and NO_2_. Double counting of mortality from co-exposure to both PM_2.5_ and NO_2_ is less likely of concern as we use the NO_2_-mortality exposure response coefficient adjusted for confounding PM_2.5_ exposure (“Quantification of adverse health outcomes” section). For cancer incidences, we assume that cancer risks are additive following the US EPA’s approach (*92*). This is likely to overestimate cancer incidences in Texas, Colorado, and North Dakota, where large concentrations of HAPs from combined upstream and midstream activities are collocated (Fig. 2, J, M, and P). The size of this overestimate is probably small, as 103 cancer incidences in these three states linked to combined upstream and midstream activities account for only 6% of the total HAPs cancer burden.

We find that on a national scale, the greatest health burden of air pollution linked to the O&G lifecycle is from end-use in densely populated states. The health burden of other lifecycle stages is most severe in states encompassing large, active extraction basins for combined upstream and midstream activities, and those with or neighboring states with intensive processing activities for downstream. Between our study year of 2017 and 2023, annual US O&G production has increased by 40% and consumption by ~8% (*4*). Absent emissions control measures, these trends suggest much larger present-day air pollutant precursor emissions from upstream, midstream, and downstream stages and relatively similar emissions from end-use, the O&G lifecycle stage accounting for >80% of the health burden. US population between 2017 and 2021 has increased by 4% for adults and 16% for the elderly and declined by 2% for children. Baseline rates of mortality have declined by 7% for elderly and increased by only 2% for adults, and those of pediatric asthma incidences increased by only 1% from 2017 to 2021 (*93*). The combined effect of these factors suggests that contemporary health burdens for end-use are likely similar to our findings, while those for upstream, midstream, and downstream may be more severe than our 2017 estimate. Disparate exposures to air pollution have also worsened with the boom in unconventional O&G extraction (*60*), and so, the 40% increase in O&G production suggests that our absolute disparities are conservative, especially for population subgroups living in close proximity to very productive wells (*42*).

We note also that the air pollutants and associated public health burdens and disparities assessed in this study are not exhaustive and do not capture the full spectrum of those arising from indoor air pollution and from acute exposure to O&G lifecycle emissions. For example, indoor air pollution from gas stoves have been linked to 13% of childhood asthma prevalence in the US (*94*), and acute and hyperlocal effects from hourly or daily spikes in air pollutant concentrations can be substantial near O&G extraction sites and oil refineries (*95*, *96*). Future studies could quantify these effects.

Despite inherent uncertainties in estimating health burdens, our findings serve to highlight the climate mitigation co-benefit of phasing out O&G and the extent to which health burdens and disparities in air pollution exposure would be alleviated. Although our health burden results are overall conservative, these provide a foundation for future studies that could further refine quantification of disparities to support civil, community, and regulatory action. These could, for example, focus on finer spatial and temporal resolution models that better resolve isolated sources such as those producing large, intermittent quantities of HAPs. Our findings also support prioritizing actions that target end-use sources for reducing the health burden of the total population, particularly in California, Texas, and New York, and downstream activities for alleviating the most severe of the relative health disparities experienced by the Black population for almost all adverse health outcomes.

## MATERIALS AND METHODS

### Hourly, gridded air pollutant emissions of individual O&G lifecycle stages

Health burden assessment of individual lifecycle stages is not feasible with the available regional inventories of gridded air pollutant emissions. The US Fuel-based Oil and Gas (FOG) inventory (*84*, *97*) is limited to a subset of air pollutant precursors specific to O&G production. In the US EPA National Emission Inventory (NEI) (*98*), O&G activities are lumped with non-O&G activities within predefined sectors such as “Storage and Transfer” that cannot be readily reassigned to individual lifecycle stages. Given this, multiple contemporary inventories are combined to construct gridded (0.1° × 0.1°), monthly air pollutant emissions for each lifecycle stage for input to GEOS-Chem.

For CH_4_ emissions only, we use the 2017 Global Fuel Exploitation Inventory (GFEI) (*70*, *99*) for upstream, midstream, and downstream stages and the 2014 Community Emissions Data System (CEDS) version 1 inventory for end-use (*100*). The CEDS CH_4_ emission year is 3 years earlier than the study year, but CH_4_ end-use emissions only account for 0.9% of the total (Fig. 1). Non-CH_4_ anthropogenic emissions are from the US Fuel-based Inventory for Vehicular Emissions (FIVE) (https://csl.noaa.gov/groups/csl7/measurements/2020covid-aqs/emissions/; last accessed 5 September 2022) for mobile sources such as on-road vehicles (*14*, *101*), the global Aviation Emissions Inventory Code (AEIC) for aviation (*102*, *103*), and CEDS version 2 for shipping (*104*). FIVE is for 2018, AEIC is only available for 2005, and CEDS v2 is for our study year 2017. All other anthropogenic emissions are from the most recent publicly available NEI emissions for 2017 (NEI 2017; https://www.epa.gov/air-emissions-inventories/2017-national-emissions-inventory-nei-data; last accessed 5 September 2022) (*98*).

NEI provides annual county-level emission totals for more than 8000 active Source Classification Codes (SCCs), so SCCs for each lifecycle stage are identified and processed following the approach of Vohra *et al.* (*58*) to yield gridded, monthly emissions. A broad categorization of these SCCs into 25 categories for O&G activities and 11 categories for non-O&G (or “Others”) activities is in table S1. Five hundred and eighty NEI SCCs are used for upstream and midstream emissions, 100 SCCs are used for downstream emissions, and 248 NEI SCCs are used for end-use emissions. The NEI SCC end-use emissions are added to FIVE, AEIC, and CEDS v2 for all the non-CH_4_ emissions. Temporal variability in emissions is achieved with hourly and day-of-week scaling factors and daylight savings time zone shifts, as described by Vohra *et al.* (*58*).

### Air pollutant modeling and validation

We use GEOS-Chem version 13.0.0 (https://doi.org/10.5281/zenodo.4618180; last accessed 1 March 2023) driven by NASA GEOS-FP meteorology to simulate ambient surface concentrations of health-damaging air pollutants linked to emissions from major O&G lifecycle stages. The model is run in a nested configuration over CONUS (23°N to 51°N, 128°W to 63.5°W) at 0.25° × 0.3125° (~28 km latitude × ~27 km longitude at the nested domain center) for 2017, with three-hourly boundary conditions from a global simulation that is at 4° × 5°. The emissions detailed in the previous section are gridded to the model horizontal resolution, and temporal scaling factors are applied via the GEOS-Chem emissions processing package, Harmonized Emissions Component (HEMCO) (*105*). The model chemistry is initialized with spin-ups of a year for the global boundary conditions and 2 months for the nested domain. The model is simulated over time periods much shorter than the lifetime of CH_4_ (~10 years), so to account for the effect of individual lifecycle stage CH_4_ emissions on O_3_ and the hydroxyl radical (OH), we generate and use CH_4_ pseudo fluxes, as in Ryan *et al.* (*106*).

To estimate surface air pollutant concentrations attributable to emissions in each lifecycle stage, we conduct a baseline simulation with all emissions as described in the previous section and three sensitivity simulations. In the sensitivity simulations, emissions in Fig. 1 from upstream and midstream activities are set to zero for the first, downstream set to zero for the second, and end-use set to zero for the third. Meteorology and boundary conditions are the same in all simulations. The difference between the baseline and each sensitivity simulation provides an estimate of the contribution of each of the lifecycle stages to air pollution in the CONUS and across the border in southern Canada and northern Mexico that are within the nested model domain. The zero-out approach we use is standard and widely adopted to quantify the contribution of targeted sources or sectors to air quality and health (*54*, *55*, *107*–*109*). Inventories of open fire and natural emissions and GEOS-Chem treatment of gas- and aerosol-phase chemistry are detailed in Vohra *et al.* (*58*).

We evaluate modeled annual mean fine particulate matter (PM_2.5_) and nitrogen dioxide (NO_2_), and peak-season MDA8 O_3_ against observations from the US EPA Air Quality System (AQS) database (https://aqs.epa.gov/aqsweb/documents/data_api.html; last accessed 1 March 2023) for 2017. We also use 2017 PM_2.5_ observations from the mostly rural network sites of the Interagency Monitoring of Protected Visual Environments (IMPROVE) program (http://vista.cira.colostate.edu/Improve/; last accessed 16 April 2023). NO_2_ is measured with chemiluminescence instruments that exhibit positive interference from thermal decomposition of NO*_x_* reservoir compounds (*110*), so we calculate an equivalent NO_2_^*^ for GEOS-Chem to compare to the observations. Calculation of modeled NO_2_^*^ and of PM_2.5_ from the individual aerosol components is described by Vohra *et al.* (*58*). Only observations with at least 75% temporal coverage in each month of interest (annual for PM_2.5_ and NO_2_, March to August for MDA8 O_3_) are gridded to the GEOS-Chem nested grid for comparison to the model (fig. S2). This filtering removes many of the sites for the MDA8 O_3_ comparison, as 982 sites have no hourly O_3_ data in March 2017. HAP measurement sites are not as densely distributed as PM_2.5_, NO_2_, and MDA8 O_3_ and are also not collocated with O&G activities. So, we rely on intermittent field campaigns targeting large, isolated sources from O&G upstream activities to evaluate our modeled HAP concentrations.

In general, the model has moderate spatial consistency (*R* ~ 0.6 to 0.7) with the surface observations. GEOS-Chem is on average only 3 to 4% more than observed NO_2_^*^ and MDA8 O_3_, but is 21% more than observed PM_2.5_. Standard major axis (SMA) regression slopes of 0.8 to 0.9 for MDA8 O_3_ and NO_2_ support the use of model perturbation simulations to determine influence of individual lifecycle stages on air pollutant surface concentrations. SMA regression statistics for PM_2.5_ are sensitive to the population of points with GEOS-Chem PM_2.5_ > 20 μg m^−3^ and observed PM_2.5_ < 15 μg m^−3^ (fig. S2B). If instead we use a Theil-Sen fit that is less influenced by outliers, the regression slope is 0.82 [95% confidence interval (CI): 0.73 to 0.91]. The intercept is ~2.8 μg m^−3^, suggesting that the model normalized mean bias (NMB) of 21% is dominated by a systematic overestimate in PM_2.5_ rather than an overestimate in the response of the model to perturbations in primary and precursors of PM_2.5_.

### Quantification of adverse health outcomes

We determine the health burden of air pollution from each major O&G lifecycle stage by estimating specific adverse health outcomes associated with O&G activities with known statistical relationships between air pollution exposure and health risk (*23*–*29*). These include PM_2.5_-attributable non-accidental mortality and preterm births, NO_2_-attributable non-accidental mortality and pediatric asthma incidences, MDA8 O_3_–attributable COPD mortality, and HAP-attributable cancer incidences. For all health outcomes, relative risks are determined using a health risk assessment model of the relationship between risk ratios and ambient surface air pollutant concentrations. To estimate the relative risk of adult (25+ years) premature mortality linked to long-term exposure to PM_2.5_, we use the health risk assessment model from Marais *et al.* (*91*) that is based on Weichenthal *et al.* (*111*) and combines two existing health risk assessment models. These are the extended Shape Constrained Health Impact Function (eSCHIF) that covers low PM_2.5_ concentrations (2.5 to 17.7 μg m^−3^) (*23*) and the Fusion model for intermediate to high (up to 83 μg m^−3^) PM_2.5_ concentrations (*112*). To estimate the incidences of preterm birth, we use the relative risk of 1.12 (95% CI: 1.06 to 1.19) per 10 μg m^−3^ increase in annual mean PM_2.5_ from Ghosh *et al.* (*26*). For long-term exposure to NO_2_, we use the relative risk of 1.046 (95% CI: 1.044 to 1.049) per 10 ppb increase in annual mean NO_2_ from Eum *et al.* (*113*) to estimate premature mortality and 1.26 (95% CI: 1.10 to 1.37) per 10 ppb increase in annual mean NO_2_ from Khreis *et al.* (*28*) to estimate asthma incidences in children (1 to 18 years old). The relative risk for NO_2_-attributable mortality is for people aged 65+ years and is adjusted for co-exposure to PM_2.5_. For peak-season exposure to O_3_ leading to premature mortality from COPD, we use the relative risk of 1.06 (95% CI: 1.03 to 1.10) per 10 ppb increase in MDA8 O_3_, derived using epidemiological data from five cohort studies by GBD 2019 (*27*). Cancer risk is quantified with the US EPA recommended upper-bound risk estimates from the Integrated Risk Information System (IRIS) of 11 in a million for formaldehyde, 2.2 in a million for acetaldehyde, and 7.8 in a million for benzene each for a lifetime exposure of 1 μg m^−3^ (*29*, *73*, *74*, *114*). Counterfactual air pollutant concentrations below which the relative risk to health is assumed to be 1 are 2.5 μg m^−3^ for PM_2.5_ (*23*, *111*), 32.4 ppb for MDA8 O_3_ (*27*, *115*), and 2 ppb for NO_2_ (*28*, *116*). No counterfactual is used for formaldehyde, acetaldehyde, and benzene, due to a lack of evidence (*117*).

The relative risks from exposure to PM_2.5_, NO_2_, and MDA8 O_3_ are converted to absolute occurrences using population or birth data and baseline rates of mortality or incidences of asthma or preterm birth. Incidences of cancer are determined by multiplying cancer risks by population. WorldPop (*118*) provides age-resolved population data for 2017 for 0 to 1 years, 1 to 5 years, and 5-year age increments thereafter at 1-km spatial resolution that we use after gridding these to the GEOS-Chem nested resolution. There are other high-spatial-resolution population datasets such as SocScape (https://socscape.edu.pl; last accessed 10 February 2025) that report populations by race and ethnicity, but these are not age-resolved and are not available for our study year. Baseline rates of non-accidental and COPD mortality and preterm birth incidences are available for each US county for 2017 from US Centers for Disease Control and Prevention Wide-ranging ONline Data for Epidemiologic Research (CDC WONDER; https://wonder.cdc.gov/mcd-icd10.html, last accessed 20 September 2024). CDC WONDER suppresses data for counties with fewer than 10 incidences or deaths. Data are suppressed for 42 of 3106 counties. For these counties, we use baseline rates at the state level. The 42 counties account for <0.02% of the US adult population, so this data substitution will have negligible impact on our estimates of non-accidental and COPD mortality. Incidences of pediatric asthma are available for each US state for 2017 from the GBD (*119*). We also estimate health outcomes for the model domain covering southern Canada and northern Mexico using GBD baseline rates of non-accidental mortality that are available at the state level for Mexico and national level for Canada. All baseline rates are applied uniformly across the county (or state) to the GEOS-Chem grid cells (0.25° × 0.3125°) that overlap with the county (or state) boundaries.

Adverse health outcomes are calculated using air pollutant surface concentrations from all four GEOS-Chem simulations (model output with all emissions as is, and three sensitivity simulations detailed in the previous section). This yields gridded health outcomes and 95% CIs due to errors in the health risk assessment model. Health outcomes attributable to exposure to air pollutants from individual lifecycle stages are then determined as the difference between baseline and individual sensitivity simulation health outcomes. The gridded outcomes are then summed up for each state. These are reported in tables S2 to S7. The UIs of these health burden estimates, also in tables S2 to S7, are calculated by first adding in quadrature the relative error in state burden totals from the baseline and the sensitivity simulations, and then multiplying this computed relative error by the state burden totals. For Texas, for example, total PM_2.5_ premature mortality from all emission sources is 14,800 (95% CI: 13,700 to 16,000) (relative uncertainty of 7 to 8%) and the PM_2.5_ premature mortality for the simulation with end-use emissions set to zero is 13,000 (95% CI: 11,900 to 14,100) (relative uncertainty of 8%). The relative uncertainty for the difference in premature mortality estimates, from adding the relative uncertainties of the individual values in quadrature, is 11%, and the UI for 1800 PM_2.5_-attributable premature deaths from exposure to end-use activities is 1600 to 2000 as reported in table S2.

### Exposure and health burden disparity analyses

We examine disparities in exposure to air pollution linked to individual O&G lifecycle stages by calculating US-wide population-weighted mean air pollutant concentrations for individual US ethnic and racial groups as defined by the US Census Bureau. For this, we use the population, age structure, and demographics in each US county from the American Community Survey (ACS) 5-year estimates for 2013–2017. The survey asks respondents to self-identify their ethnicity as “Hispanic or Latino” or “Not Hispanic or Latino” and to self-identify their race as single or mixed. In our analysis, we consider the non-Hispanic “single-race” populations of “American Indian or Alaska Native,” “Asian,” “Black or African American,” and “Native Hawaiian or Other Pacific Islander.” This excludes just 3% of the ACS respondents that identify as multiracial. We refer to the American Indian or Alaska Native population as “Native American” and further combine Native Hawaiian or Other Pacific Islander with the Asian population, as in Geldsetzer *et al.* (*76*), because the health data from CDC WONDER for these two population subgroups are lumped together as “Asian and Other Pacific Islander.” “White” groups are also included for comparative purposes. To calculate the population count in each model grid by race and ethnicity, the GEOS-Chem grid (0.25° × 0.3125°) is intersected with the shapefiles of US counties and the population count of each demographic group in a county is weighted by the area of overlap with the intersecting model grid cells. This ensures that population and air pollutant concentrations linked to individual O&G lifecycle stages from the “Air pollutant modeling and validation” section are on the same grid before calculating national population-weighted mean air pollutant concentrations for different demographic (ethnic and racial) groups.

We assess racial and ethnic disparities in health burdens by comparing population-standardized rates of premature mortality and incidences of childhood asthma, preterm births, and cancer across all O&G lifecycle stages and the selected demographic groups. For this, we use age-standardized baseline mortality rates in each county by demographic group from CDC WONDER. The use of age-standardized rates ensures consistent comparison across demographic groups with different age distributions by accounting for differing susceptibilities to health outcomes across racial-ethnic groups, age groups, and locations (*120*, *121*). For data unavailable for specific demographic groups, we use the baseline rates for the total population in that county. Depending on the racial-ethnic population subgroup, this gap filling is done for up to 1426 of 3106 CONUS counties for non-accidental mortality (fig. S4) and up to 959 counties for COPD mortality. Although data gap filling is required for many counties, it is mostly only for counties with few people in the targeted racial-ethnic subgroup population: <5% of the national subgroup population for Asian, Black, Hispanic, and white populations, limiting the impact on absolute and relative health burdens. Gap filling is greater (20%) for the Native American population (fig. S4). If we artificially increase or decrease the state-level baseline mortality rates (BMRs) in the 1129 gap-filled counties by 20% to represent potential biases introduced by this gap filling, the magnitude of the relative disparities changes, but the relative order of disparate health burdens across the racial-ethnic subgroups does not change. A 20% increase increases Native American relative disparities in premature mortality from exposure to PM_2.5_ from 1.26 to 1.34 for combined upstream and midstream, 0.60 to 0.66 for downstream, and 0.69 to 0.74 for end-use, but the ranking relative to other subpopulation groups is unchanged. Similarly, findings are unchanged with a more extreme 30% bias test. If data are unavailable for the total population, we use rates reported for the entire state. State-wide data are used for 38 counties for non-accidental mortality and 844 counties for COPD mortality.

PM_2.5_-, NO_2_-, and MDA8 O_3_–attributable mortality are estimated as described in the “Quantification of adverse health outcomes” section using the demographic-specific baseline mortality rates. These are then divided by the total US population of the population subgroup to obtain premature mortality rates for each population subgroup. CDC WONDER does not provide baseline rates of childhood asthma by racial and ethnic groups, so instead we rely on the state total incidence rates from GBD to calculate NO_2_-attributable childhood asthma. This leads to near-identical patterns in exposure and burden disparities linked to NO_2_. Similarly, the cancer incidences are mapped to population, so patterns in exposure and burden disparities linked to HAPs are also identical.
