# Supplementary material for: The health burden and racial-ethnic disparities of air pollution from the major oil and gas lifecycle stages in the United States
Source: Sci Adv. 2025 Aug 22;11(34):eadu2241. doi: 10.1126/sciadv.adu2241 (PMC12372900; doi:10.1126/sciadv.adu2241)
Supplement: Supplementary file 1 — Figs. S1 to S4 Tables S1 to S8 [file sciadv.adu2241_sm.pdf]

Science Advances
AAAS

Supplementary Materials for

The health burden and racial-ethnic disparities of air pollution from the major oil and gas lifecycle stages in the United States

Karn Vohra *et al.*

Corresponding author: Eloise A. Marais, e.marais@ucl.ac.uk

Sci. Adv. **11**, eadu2241 (2025)
DOI: 10.1126/sciadv.adu2241

This PDF file includes:

Figs. S1 to S4
Tables S1 to S8

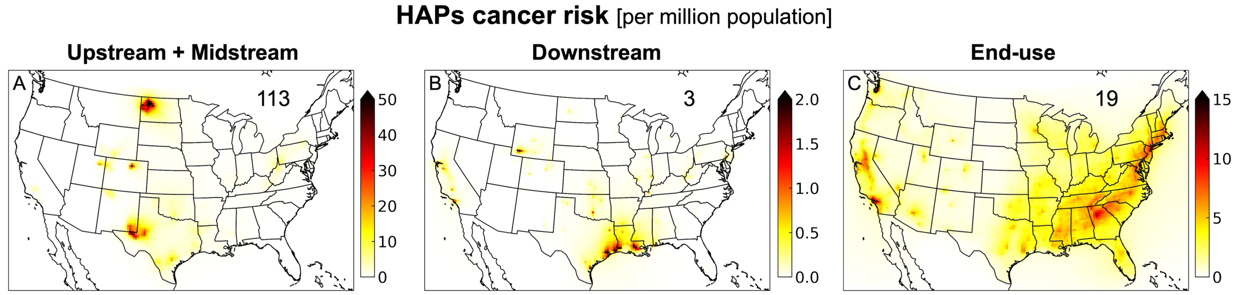

Supplementary Figure 1. Gridded cancer risks of O&G lifecycle stage hazardous air pollutants (HAPs). Maps are the sum of individual cancer risks from lifetime exposure to formaldehyde, acetaldehyde, and benzene. Inset values give the maximum risk value for each stage, as the colour bar saturates. Note that colorscales differ.

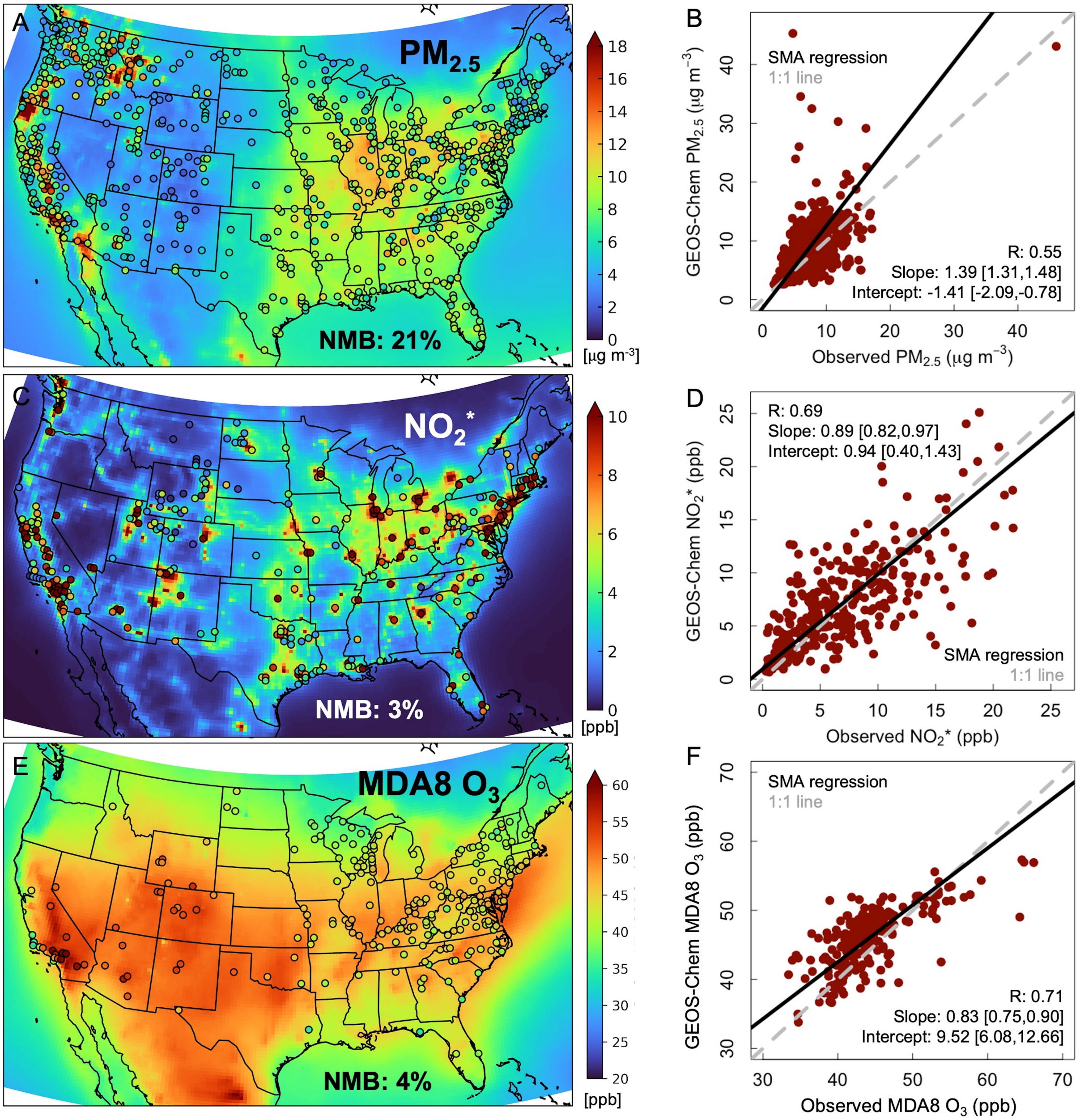

Supplementary Figure 2. Assessment of GEOS-Chem contiguous US surface $PM_{2.5}$, NO_2^*, and MDA8 O_3 in 2017. Maps compare simulated (background) and observed (circles) annual mean $PM_{2.5}$ (A) and NO_2^* (C), and spring-summer mean MDA8 O_3 (E). Values inset are the model normalized mean bias (NMB) for coincident grid cells and observations. Scatter plots compare coincident modelled and observed $PM_{2.5}$ (B), NO_2^* (D) and MDA8 O_3 (F). Lines are the standard major axis (SMA) regression (black solid) and 1:1 agreement (grey dashed). Values inset are Pearson's correlation coefficient (R) and SMA regression statistics. Values in square brackets are slope and intercept 95% confidence intervals (CI).

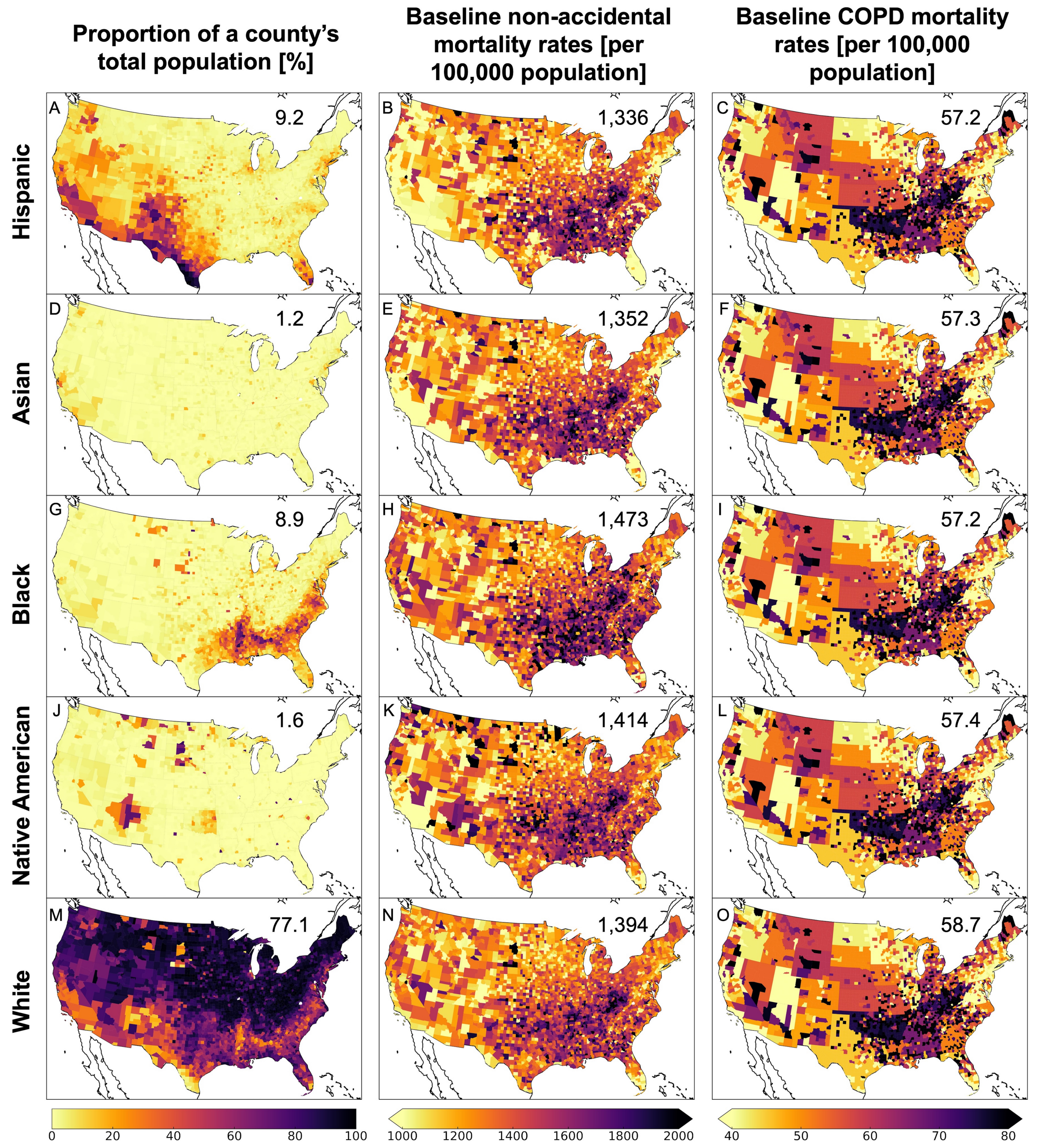

Supplementary Figure 3. US county-level demographic and health data for 2017. Maps show proportion of total county population (left column), age-standardized baseline rates of non-accidental mortality (center) and baseline rates of COPD mortality (right) for Hispanic, Asian, Black, Native American and White population subgroups. Demographic data are from ACS and health data are from CDC WONDER (Methods Section 4). Values inset are CONUS means. CONUS mean baseline mortality rate is 1,397 per 100,000 people for non-accidental mortality and 57.4 per 100,000 people for COPD mortality.

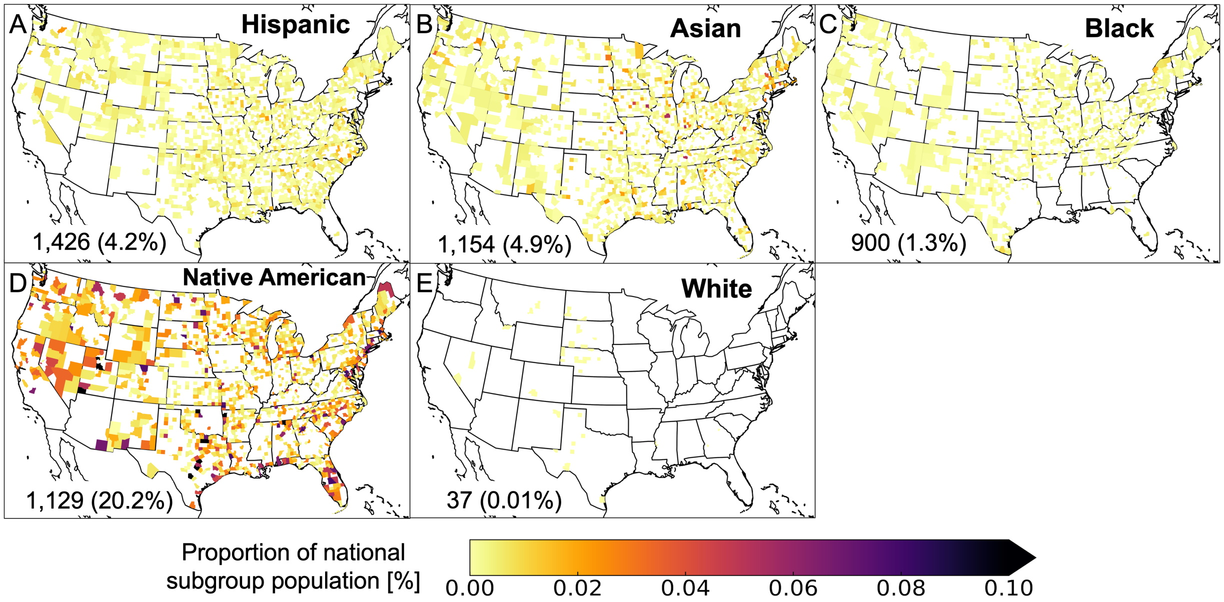

Supplementary Figure 4. US counties with suppressed all-cause mortality rates. Maps show proportion of total national population of Hispanic (A), Asian (B), Black (C), Native American (D) and White (E) population subgroups by county for counties with suppressed CDC WONDER health data. Demographic data are from ACS (Methods Section 4). Values inset are the number of counties with suppressed all-cause mortality rates and the total proportion of national subgroup population in these counties is in brackets.

Supplementary Table 1. Broad categories of US National Emissions Inventory (NEI) Source Classification Codes (SCCs) assigned to O&G lifecycle stages and other activities

Category	NEI sources and processes
Upstream	Oil and Gas Production (Flares, On-site Tanks, On-Site Fugitive Emissions)
	On-site Gas Compression
	Oil and Gas Exploration, Drilling
	Offshore Oil and Gas Production
Midstream	Natural Gas Transmission and Storage Facilities
	Industrial Fuel Storage Tanks
	Petroleum Storage at Refineries
	Petroleum Bulk Terminal Storage
	Oil and Gas Field Storage
	Fuel Tanker Trucks, Rail Tank Cars and Tanker Marine Vessels (Fuel Evaporation)
	Gas Stations, Other Vehicle Refuelling
	Pipeline Transportation
	Compressor Stations
Downstream	Petroleum Refineries
	Natural Gas Processing Plants
	Asphalt Manufacturing
End-Use	Gas-powered and Oil-powered Electricity Generation Units
	Gas-fired and Oil-fired Boilers (Industrial, Commercial)
	Gas-fired and Oil-Fired Heating in Residential, Commercial, Industrial Buildings (includes stoves)
	Gas-powered and Oil-powered Engines (Industrial, Commercial)
	Industrial Flares
	Airport Equipment
	Commercial Marine Vessels
	Locomotives
	On-road and Non-Road Vehicles
Others	Coal-Powered and Biomass-powered Electricity Generation Units
	Coal-Fired and Biomass-fired Boilers (Industrial, Commercial)
	Chemical Manufacturing
	Metal Manufacturing
	Other Manufacturing (Electronics, Automotive, Paper, Textiles, Food, etc.)
	Chemical Storage
	Solid and Liquid Waste Disposal/Processing
	Agriculture
	Coal and Biomass-Fired Building Heating (Residential, Commercial, Industrial)
	Construction
	Mining

Supplementary Table 2. $PM_{2.5}$-attributable adult (25+ years) non-accidental premature mortality linked to major O&G lifecycle stages in 2017[ǂ,*].

State	Upstream + Midstream [UI]	Downstream [UI]	End-use [UI]	Lifecycle Total [UI]	Lifecycle PAF[‖] [UI]
Alabama	22 [20-25]	35 [31-38]	522 [471-577]	579 [522-640]	1.2 [1.1-1.4]
Arizona	5 [4-5]	0 [0-0]	618 [527-705]	623 [531-710]	1.1 [1-1.3]
Arkansas	23 [21-25]	33 [30-36]	251 [228-278]	307 [279-339]	1.1 [1-1.2]
California	123 [103-143]	374 [314-435]	9,525 [8,113-10,943]	10,022 [8,530-11,521]	4.2 [3.5-4.8]
Colorado	31 [24-37]	12 [9-14]	380 [286-468]	423 [319-519]	1.2 [0.9-1.5]
Connecticut	3 [3-3]	2 [2-2]	587 [510-661]	592 [515-666]	2.1 [1.8-2.4]
Delaware	5 [4-5]	10 [9-11]	182 [162-203]	197 [175-219]	2.4 [2.1-2.6]
District of Columbia	1 [1-2]	1 [1-2]	98 [88-110]	100 [90-114]	2.3 [2.1-2.6]
Florida	8 [8-9]	16 [14-18]	1,022 [904-1,136]	1,046 [926-1,163]	0.6 [0.5-0.6]
Georgia	37 [34-42]	49 [45-55]	1,174 [1,058-1,300]	1,260 [1,137-1,397]	1.7 [1.5-1.9]
Idaho	0 [0-0]	0 [0-0]	63 [51-73]	63 [51-73]	0.5 [0.4-0.6]
Illinois	47 [41-54]	69 [60-78]	2,044 [1,810-2,304]	2,160 [1,911-2,436]	2.2 [1.9-2.5]
Indiana	36 [33-41]	27 [25-30]	1,140 [1,031-1,269]	1,203 [1,089-1,340]	2.1 [1.9-2.4]
Iowa	16 [14-17]	6 [6-7]	291 [263-321]	313 [283-345]	1.2 [1.1-1.3]
Kansas	29 [26-32]	19 [18-22]	213 [190-236]	261 [234-290]	1.1 [1-1.3]
Kentucky	28 [25-31]	23 [20-25]	676 [610-752]	727 [655-808]	1.7 [1.5-1.9]
Louisiana	21 [19-23]	141 [127-157]	305 [275-338]	467 [421-518]	1.2 [1.1-1.3]
Maine	1 [1-1]	1 [1-1]	130 [101-155]	132 [103-157]	1 [0.8-1.2]
Maryland	25 [23-28]	23 [20-25]	1,090 [979-1,215]	1,138 [1,022-1,268]	2.5 [2.2-2.7]
Massachusetts	6 [5-6]	2 [2-2]	872 [755-983]	880 [762-991]	1.7 [1.5-1.9]
Michigan	45 [40-50]	21 [19-24]	1,389 [1,243-1,547]	1,455 [1,302-1,621]	1.7 [1.5-1.9]
Minnesota	11 [10-12]	3 [3-3]	466 [416-518]	480 [429-533]	1.3 [1.2-1.4]
Mississippi	15 [13-16]	36 [32-39]	231 [208-255]	282 [253-310]	1 [0.9-1.1]
Missouri	39 [35-43]	26 [24-30]	697 [626-779]	762 [685-852]	1.4 [1.3-1.6]
Montana	1 [1-1]	0 [0-0]	23 [19-28]	24 [20-29]	0.3 [0.2-0.3]
Nebraska	13 [11-14]	5 [4-6]	111 [98-125]	129 [113-145]	0.9 [0.8-1]
Nevada	1 [1-1]	1 [1-1]	336 [259-401]	338 [261-403]	1.5 [1.1-1.7]
New Hampshire	1 [1-1]	1 [1-1]	149 [121-172]	151 [123-174]	1.4 [1.1-1.6]
New Jersey	30 [26-34]	45 [40-51]	2,181 [1,921-2,456]	2,256 [1,987-2,541]	3.2 [2.9-3.7]

State	Upstream + Midstream [UI]	Downstream [UI]	End-use [UI]	Lifecycle Total [UI]	Lifecycle PAF[‖] [UI]
New Mexico	21 [15-26]	2 [2-3]	126 [85-163]	149 [102-192]	0.9 [0.6-1.1]
New York	60 [52-68]	50 [43-57]	3,638 [3,166-4,118]	3,748 [3,261-4,243]	2.6 [2.3-3]
North Carolina	22 [20-24]	15 [14-17]	1,103 [977-1,230]	1,140 [1,011-1,271]	1.4 [1.2-1.5]
North Dakota	2 [2-2]	0 [0-0]	13 [11-14]	15 [13-16]	0.3 [0.2-0.3]
Ohio	81 [74-91]	38 [34-42]	2,250 [2,035-2,505]	2,369 [2,143-2,638]	2.2 [2-2.4]
Oklahoma	49 [45-55]	34 [31-38]	332 [300-368]	415 [376-461]	1.2 [1.1-1.3]
Oregon	0 [0-0]	0 [0-0]	380 [327-433]	380 [327-433]	1.2 [1-1.3]
Pennsylvania	87 [77-98]	111 [98-125]	3,390 [3,027-3,795]	3,588 [3,202-4,018]	3 [2.7-3.3]
Rhode Island	0 [0-0]	0 [0-0]	140 [117-160]	140 [117-160]	1.6 [1.3-1.8]
South Carolina	15 [13-16]	23 [21-26]	552 [496-612]	590 [530-654]	1.4 [1.2-1.5]
South Dakota	3 [3-4]	1 [0-1]	30 [24-36]	34 [27-41]	0.5 [0.4-0.6]
Tennessee	31 [28-35]	34 [31-38]	846 [764-939]	911 [823-1,012]	1.5 [1.3-1.7]
Texas	238 [213-266]	302 [271-337]	1,812 [1,614-2,020]	2,352 [2,098-2,623]	1.3 [1.2-1.5]
Utah	4 [3-4]	1 [1-1]	211 [165-252]	216 [169-257]	1.4 [1.1-1.6]
Vermont	1 [0-1]	0 [0-0]	58 [46-68]	59 [46-69]	1.1 [0.9-1.3]
Virginia	21 [19-24]	10 [9-11]	943 [838-1,051]	974 [866-1,086]	1.6 [1.4-1.8]
Washington	0 [0-0]	0 [0-0]	864 [751-973]	864 [751-973]	1.8 [1.5-2]
West Virginia	17 [15-19]	9 [8-10]	242 [215-269]	268 [238-298]	1.3 [1.1-1.4]
Wisconsin	18 [16-20]	4 [3-4]	572 [512-636]	594 [531-660]	1.3 [1.2-1.4]
Wyoming	2 [1-3]	1 [0-1]	35 [18-52]	38 [19-56]	0.9 [0.4-1.3]
Contiguous US	1,294 [1,143-1,458]	1,616 [1,427-1,821]	44,301 [38,994-49,837]	47,211 [41,564-53,116]	1.9 [1.7-2.1]

[ǂ] State totals are rounded to nearest whole numbers. Contiguous US totals are rounded after summing unrounded state values.

*Uncertainty interval for health burden estimate. Calculation described in Methods Section 3.

[‖]Population Attributable Fraction (PAF) or fraction of total adult (25+ years) non-accidental mortality attributable to $PM_{2.5}$ from the O&G lifecycle. Expressed as a percentage.

Supplementary Table 3. NO_2-attributable adult (65+ years) non-accidental premature mortality linked to major O&G lifecycle stages in 2017[ǂ,*].

State	Upstream + Midstream [UI]	Downstream [UI]	End-use [UI]	Lifecycle Total [UI]	Lifecycle PAF[‖] [UI]
Alabama	10 [9-11]	4 [4-4]	276 [260-301]	290 [273-316]	0.8 [0.7-0.8]
Arizona	1 [1-2]	0 [0-0]	1,190 [1,119-1,295]	1,191 [1,120-1,297]	2.6 [2.5-2.9]
Arkansas	15 [14-16]	1 [1-1]	150 [141-163]	166 [156-180]	0.7 [0.7-0.8]
California	27 [25-29]	74 [70-81]	7,172 [6,749-7,804]	7,273 [6,844-7,914]	3.6 [3.4-3.9]
Colorado	53 [50-58]	4 [4-5]	535 [503-582]	592 [557-645]	2.2 [2-2.4]
Connecticut	1 [1-1]	1 [1-1]	479 [451-522]	481 [453-524]	1.9 [1.8-2.1]
Delaware	1 [1-1]	2 [2-3]	124 [117-135]	127 [120-139]	1.8 [1.7-2]
District of Columbia	1 [1-1]	0 [0-0]	95 [89-103]	96 [90-104]	3.8 [3.5-4.1]
Florida	-1 [-1--1]	1 [1-1]	972 [914-1,059]	972 [914-1,059]	0.6 [0.6-0.7]
Georgia	2 [2-2]	1 [1-1]	1,021 [960-1,112]	1,024 [963-1,115]	1.7 [1.6-1.9]
Idaho	0 [0-0]	0 [0-0]	58 [54-63]	58 [54-63]	0.5 [0.5-0.6]
Illinois	22 [21-24]	17 [16-19]	1,786 [1,680-1,945]	1,825 [1,717-1,988]	2.2 [2-2.4]
Indiana	16 [15-17]	2 [2-3]	817 [768-889]	835 [785-909]	1.8 [1.6-1.9]
Iowa	2 [2-2]	0 [0-1]	208 [195-226]	210 [197-229]	0.9 [0.9-1]
Kansas	36 [34-39]	5 [5-6]	214 [201-233]	255 [240-278]	1.3 [1.2-1.4]
Kentucky	22 [21-24]	1 [1-2]	432 [406-470]	455 [428-496]	1.4 [1.3-1.5]
Louisiana	27 [26-30]	87 [82-95]	306 [288-334]	420 [396-459]	1.4 [1.3-1.5]
Maine	0 [0-0]	0 [0-0]	33 [31-35]	33 [31-35]	0.3 [0.3-0.3]
Maryland	9 [9-10]	2 [2-2]	974 [916-1,061]	985 [927-1,073]	2.6 [2.5-2.9]
Massachusetts	1 [1-1]	0 [0-1]	805 [771-855]	806 [772-857]	1.8 [1.7-1.9]
Michigan	13 [12-14]	1 [1-1]	1,136 [1,069-1,237]	1,150 [1,082-1,252]	1.6 [1.5-1.8]
Minnesota	2 [2-2]	1 [1-1]	565 [531-615]	568 [534-618]	1.7 [1.6-1.8]
Mississippi	3 [3-3]	4 [3-4]	79 [74-86]	86 [80-93]	0.4 [0.4-0.4]
Missouri	15 [14-17]	4 [4-4]	573 [539-624]	592 [557-645]	1.3 [1.2-1.4]
Montana	0 [0-0]	0 [0-0]	11 [10-12]	11 [10-12]	0.1 [0.1-0.2]
Nebraska	4 [3-4]	0 [0-0]	93 [87-101]	97 [90-105]	0.8 [0.7-0.8]
Nevada	0 [0-0]	0 [0-0]	489 [460-532]	489 [460-532]	2.5 [2.4-2.7]
New Hampshire	0 [0-0]	0 [0-0]	71 [68-75]	71 [68-75]	0.7 [0.7-0.8]
New Jersey	9 [8-9]	13 [12-14]	2,015 [1,896-2,193]	2,037 [1,916-2,216]	3.5 [3.3-3.8]

State	Upstream + Midstream [UI]	Downstream [UI]	End-use [UI]	Lifecycle Total [UI]	Lifecycle PAF[‖] [UI]
New Mexico	12 [12-14]	0 [0-0]	158 [149-172]	170 [161-186]	1.2 [1.2-1.3]
New York	30 [28-33]	6 [6-7]	3,212 [3,022-3,496]	3,248 [3,056-3,536]	2.7 [2.6-3]
North Carolina	5 [4-5]	1 [0-1]	731 [687-796]	737 [691-802]	1.1 [1-1.2]
North Dakota	2 [2-2]	0 [0-0]	16 [15-17]	18 [17-19]	0.4 [0.4-0.4]
Ohio	50 [47-54]	9 [8-9]	1,565 [1,472-1,705]	1,624 [1,527-1,768]	1.8 [1.7-2]
Oklahoma	62 [58-67]	11 [10-12]	402 [378-438]	475 [446-517]	1.7 [1.6-1.8]
Oregon	0 [0-0]	0 [0-0]	376 [354-410]	376 [354-410]	1.3 [1.3-1.5]
Pennsylvania	164 [154-178]	32 [30-34]	2,299 [2,162-2,503]	2,495 [2,346-2,715]	2.4 [2.2-2.6]
Rhode Island	0 [0-0]	0 [0-0]	104 [100-111]	104 [100-111]	1.3 [1.3-1.4]
South Carolina	1 [1-1]	1 [1-2]	316 [297-345]	318 [299-348]	0.9 [0.8-1]
South Dakota	0 [0-0]	0 [0-0]	15 [14-16]	15 [14-16]	0.3 [0.3-0.3]
Tennessee	9 [8-10]	1 [1-1]	542 [509-590]	552 [518-601]	1.1 [1-1.2]
Texas	310 [292-338]	118 [111-129]	2,659 [2,501-2,896]	3,087 [2,904-3,363]	2.2 [2.1-2.4]
Utah	3 [3-3]	0 [0-0]	359 [337-390]	362 [340-393]	2.7 [2.5-2.9]
Vermont	0 [0-0]	0 [0-0]	5 [5-5]	5 [5-5]	0.1 [0.1-0.1]
Virginia	12 [12-13]	1 [1-1]	708 [665-771]	721 [678-785]	1.4 [1.3-1.5]
Washington	0 [0-0]	0 [0-0]	848 [797-923]	848 [797-923]	2 [1.9-2.2]
West Virginia	48 [45-52]	3 [3-3]	148 [139-161]	199 [187-216]	1.2 [1.1-1.3]
Wisconsin	1 [1-2]	1 [1-1]	486 [457-529]	488 [459-532]	1.2 [1.1-1.3]
Wyoming	2 [2-2]	0 [0-0]	10 [9-11]	12 [11-13]	0.3 [0.3-0.4]
Contiguous US	1,003 [944-1,092]	412 [388-449]	37,635 [35,400-40,974]	39,050 [36,732-42,515]	1.9 [1.8-2]

[ǂ] State totals are rounded to nearest whole numbers. Contiguous US totals are rounded after summing unrounded state values.

*Uncertainty interval for health burden estimate. Calculation described in Methods Section 3.

[‖]Population Attributable Fraction (PAF) or fraction of total elderly (65+ years) non-accidental mortality attributable to $PM_{2.5}$ from the O&G lifecycle. Expressed as a percentage.

Supplementary Table 4. MDA8 O_3-attributable all ages chronic respiratory disease mortality linked to major O&G lifecycle stages in 2017[‡,*].

State	Upstream + Midstream [UI]	Downstream [UI]	End-use [UI]	Lifecycle Total [UI]	Lifecycle PAF[||] [UI]
Alabama	8 [2-14]	3 [1-5]	149 [38-256]	160 [41-275]	4.8 [1.2-8.3]
Arizona	4 [1-7]	0 [0-0]	112 [30-190]	116 [31-197]	3.1 [0.8-5.3]
Arkansas	14 [4-25]	3 [1-4]	88 [23-150]	105 [28-179]	4.5 [1.2-7.7]
California	8 [2-13]	-5 [-8-(-1)][±]	134 [35-228]	137 [29-240]	1.1 [0.2-1.9]
Colorado	11 [3-18]	0 [0-0]	38 [10-65]	49 [13-83]	2.1 [0.6-3.5]
Connecticut	2 [0-3]	0 [0-0]	42 [11-72]	44 [11-75]	3.2 [0.8-5.5]
Delaware	1 [0-2]	0 [0-0]	22 [6-37]	23 [6-39]	4.6 [1.2-7.7]
District of Columbia	0 [0-0]	0 [0-0]	5 [1-8]	5 [1-8]	5.1 [1-8.2]
Florida	9 [2-15]	2 [1-4]	308 [79-531]	319 [82-550]	2.7 [0.7-4.6]
Georgia	8 [2-13]	2 [1-3]	252 [65-432]	262 [68-448]	5.7 [1.5-9.8]
Idaho	0 [0-0]	0 [0-0]	11 [3-19]	11 [3-19]	1.3 [0.3-2.2]
Illinois	14 [4-24]	1 [0-3]	116 [30-199]	131 [34-226]	2.5 [0.7-4.4]
Indiana	12 [3-20]	1 [0-2]	119 [31-203]	132 [34-225]	3.3 [0.9-5.7]
Iowa	6 [2-11]	0 [0-1]	34 [9-59]	40 [11-71]	2.4 [0.7-4.2]
Kansas	13 [3-21]	1 [0-1]	41 [11-70]	55 [14-92]	3.5 [0.9-5.8]
Kentucky	11 [3-19]	1 [0-2]	127 [33-217]	139 [36-238]	4.3 [1.1-7.4]
Louisiana	10 [3-17]	10 [3-17]	87 [22-151]	107 [28-185]	4.7 [1.2-8.1]
Maine	1 [0-1]	0 [0-0]	17 [4-29]	18 [4-30]	2 [0.4-3.3]
Maryland	6 [1-9]	0 [0-0]	85 [22-146]	91 [23-155]	4.7 [1.2-8.1]
Massachusetts	3 [1-5]	0 [0-0]	71 [18-123]	74 [19-128]	2.9 [0.8-5.1]
Michigan	9 [2-16]	1 [0-1]	106 [27-183]	116 [29-200]	2.3 [0.6-4]
Minnesota	3 [1-6]	0 [0-0]	24 [6-42]	27 [7-48]	1.3 [0.3-2.3]
Mississippi	6 [2-10]	4 [1-7]	81 [21-139]	91 [24-156]	4.8 [1.3-8.2]
Missouri	18 [5-30]	2 [0-3]	109 [29-187]	129 [34-220]	3.6 [1-6.2]
Montana	0 [0-0]	0 [0-0]	6 [2-10]	6 [2-10]	0.9 [0.3-1.5]
Nebraska	5 [1-9]	0 [0-0]	15 [4-25]	20 [5-34]	2 [0.5-3.4]
Nevada	1 [0-2]	0 [0-0]	50 [13-84]	51 [13-86]	3.2 [0.8-5.4]
New Hampshire	1 [0-1]	0 [0-0]	17 [4-29]	18 [4-30]	2.7 [0.6-4.5]
New Jersey	6 [2-10]	0 [0-0]	95 [25-163]	101 [27-173]	3.5 [0.9-5.9]
New Mexico	5 [1-9]	0 [0-0]	18 [5-30]	23 [6-39]	2.1 [0.6-3.6]
New York	12 [3-21]	1 [0-1]	153 [39-262]	166 [42-284]	2.6 [0.7-4.4]
North Carolina	11 [3-18]	1 [0-2]	273 [71-468]	285 [74-488]	5.5 [1.4-9.4]
North Dakota	0 [0-1]	0 [0-0]	1 [0-2]	1 [0-3]	0.4 [0-1.2]
Ohio	18 [5-31]	1 [0-2]	207 [54-354]	226 [59-387]	3.5 [0.9-5.9]

State	Upstream + Midstream [UI]	Downstream [UI]	End-use [UI]	Lifecycle Total [UI]	Lifecycle PAF[||] [UI]
Oklahoma	27 [7-45]	2 [0-3]	105 [28-179]	134 [35-227]	4.9 [1.3-8.2]
Oregon	0 [0-0]	0 [0-0]	16 [4-27]	16 [4-27]	0.9 [0.2-1.5]
Pennsylvania	22 [6-38]	1 [0-1]	203 [53-348]	226 [59-387]	3.8 [1-6.4]
Rhode Island	1 [0-1]	0 [0-0]	14 [4-24]	15 [4-25]	3.2 [0.8-5.3]
South Carolina	4 [1-7]	1 [0-1]	145 [38-249]	150 [39-257]	5.4 [1.4-9.3]
South Dakota	1 [0-2]	0 [0-0]	4 [1-7]	5 [1-9]	1.2 [0.2-2.1]
Tennessee	13 [3-22]	2 [1-3]	207 [54-354]	222 [58-379]	5.1 [1.3-8.7]
Texas	75 [20-127]	13 [3-22]	313 [82-534]	401 [105-683]	4.2 [1.1-7.1]
Utah	1 [0-1]	0 [0-0]	14 [4-24]	15 [4-25]	1.9 [0.5-3.2]
Vermont	0 [0-1]	0 [0-0]	6 [2-11]	6 [2-12]	2 [0.7-4.1]
Virginia	9 [2-16]	0 [0-1]	141 [37-242]	150 [39-259]	4.8 [1.3-8.4]
Washington	0 [0-0]	0 [0-0]	0 [0-0]	0 [0-0]	0 [0-0]
West Virginia	10 [3-17]	0 [0-1]	59 [15-102]	69 [18-120]	4.4 [1.1-7.6]
Wisconsin	4 [1-8]	0 [0-1]	42 [11-73]	46 [12-82]	1.8 [0.5-3.3]
Wyoming	1 [0-1]	0 [0-0]	5 [1-8]	6 [1-9]	1.7 [0.3-2.5]
Contiguous US	405 [107-689]	50 [13-85]	4,292 [1,119-7,333]	4,747 [1,239-8,107]	3.3 [0.9-5.6]

ǂ State totals rounded to nearest whole numbers. Contiguous US values are rounded after summing unrounded state values.

*Uncertainty interval for health burden estimate. Calculation described in Methods Section 3.

||Population Attributable Fraction (PAF) or fraction of total all ages chronic respiratory disease mortality linked to MDA8 O_3 from the O&G lifecycle. Expressed as a percentage.

± 5 fewer premature deaths in California linked to downstream MDA8 O_3 that result from non-linear O_3 chemistry. There is a decline in O_3 concentrations of ~0.2 ppb in Los Angeles attributable to downstream activities. The large emissions of NO_x from these activities titrate away (decrease) O_3 concentrations.

Supplementary Table 5. $PM_{2.5}$-attributable preterm birth incidences linked to major O&G lifecycle stages in 2017[ǂ,*].

State	Upstream + Midstream [UI]	Downstream [UI]	End-use [UI]	Lifecycle Total [UI]	Lifecycle PAF[||] [UI]
Alabama	4 [1-7]	6 [2-9]	115 [38-195]	125 [41-211]	1.8 [0.6-3.1]
Arizona	2 [1-3]	0 [0-1]	157 [52-268]	159 [53-272]	2 [0.7-3.5]
Arkansas	4 [1-7]	5 [2-8]	53 [18-90]	62 [21-105]	1.5 [0.5-2.5]
California	30 [10-50]	83 [29-137]	2,206 [755-3,677]	2,319 [794-3,864]	5.7 [1.9-9.4]
Colorado	6 [2-10]	3 [1-5]	79 [26-136]	88 [29-151]	1.6 [0.5-2.7]
Connecticut	1 [0-1]	0 [0-1]	120 [40-205]	121 [40-207]	3.6 [1.2-6.2]
Delaware	1 [0-1]	1 [0-2]	36 [12-62]	38 [12-65]	3.5 [1.1-5.9]
District of Columbia	0 [0-1]	0 [0-0]	30 [10-51]	30 [10-52]	3.6 [1.2-6.3]
Florida	2 [1-4]	4 [1-6]	233 [76-398]	239 [78-408]	1.1 [0.4-1.9]
Georgia	7 [2-12]	8 [3-14]	354 [117-600]	369 [122-626]	2.5 [0.8-4.3]
Idaho	0 [0-0]	0 [0-0]	15 [5-26]	15 [5-26]	0.8 [0.3-1.3]
Illinois	10 [3-17]	14 [5-23]	405 [137-678]	429 [145-718]	2.8 [0.9-4.7]
Indiana	6 [2-10]	3 [1-5]	203 [68-342]	212 [71-357]	2.7 [0.9-4.6]
Iowa	3 [1-5]	1 [0-1]	57 [19-98]	61 [20-104]	1.9 [0.6-3.2]
Kansas	6 [2-11]	4 [1-7]	49 [16-83]	59 [19-101]	1.7 [0.6-3]
Kentucky	5 [2-8]	3 [1-5]	131 [44-221]	139 [47-234]	2.4 [0.8-4]
Louisiana	6 [2-10]	36 [12-62]	91 [30-155]	133 [44-227]	1.8 [0.6-3.1]
Maine	0 [0-0]	0 [0-0]	16 [5-27]	16 [5-27]	1.5 [0.5-2.6]
Maryland	4 [1-7]	2 [1-4]	241 [80-407]	247 [82-418]	3.4 [1.1-5.8]
Massachusetts	1 [0-2]	1 [0-1]	177 [58-302]	179 [58-305]	3 [1-5.2]
Michigan	6 [2-10]	2 [1-4]	261 [87-443]	269 [90-457]	2.5 [0.8-4.3]
Minnesota	3 [1-6]	1 [0-2]	117 [39-199]	121 [40-207]	2.1 [0.7-3.6]
Mississippi	3 [1-6]	8 [2-13]	65 [21-110]	76 [24-129]	1.6 [0.5-2.7]
Missouri	7 [2-12]	4 [1-7]	122 [41-205]	133 [44-224]	1.8 [0.6-3.1]
Montana	0 [0-0]	0 [0-0]	3 [1-6]	3 [1-6]	0.3 [0.1-0.6]
Nebraska	3 [1-5]	1 [0-2]	31 [10-53]	35 [11-60]	1.4 [0.5-2.5]
Nevada	0 [0-1]	0 [0-1]	86 [28-148]	86 [28-150]	2.1 [0.7-3.6]
New Hampshire	0 [0-0]	0 [0-0]	24 [8-41]	24 [8-41]	2.4 [0.8-4.2]
New Jersey	4 [1-7]	8 [3-13]	429 [145-721]	441 [149-741]	4.7 [1.6-7.8]
New Mexico	3 [1-5]	0 [0-1]	17 [6-29]	20 [7-35]	0.8 [0.3-1.4]
New York	8 [3-13]	8 [3-13]	810 [273-1,359]	826 [279-1,385]	4.2 [1.4-7]
North Carolina	5 [2-9]	3 [1-6]	286 [94-487]	294 [97-502]	2.4 [0.8-4]
North Dakota	0 [0-1]	0 [0-0]	4 [1-6]	4 [1-7]	0.5 [0.1-0.9]

State	Upstream + Midstream [UI]	Downstream [UI]	End-use [UI]	Lifecycle Total [UI]	Lifecycle PAF[‖] [UI]
Ohio	13 [4-21]	5 [2-9]	373 [125-631]	391 [131-661]	2.9 [1-5]
Oklahoma	11 [4-19]	7 [2-11]	80 [26-135]	98 [32-165]	1.8 [0.6-3.1]
Oregon	0 [0-0]	0 [0-0]	70 [23-120]	70 [23-120]	1.9 [0.6-3.3]
Pennsylvania	11 [4-18]	17 [6-28]	487 [163-822]	515 [173-868]	4.1 [1.4-6.9]
Rhode Island	0 [0-0]	0 [0-0]	25 [8-43]	25 [8-43]	3 [0.9-5.1]
South Carolina	2 [1-4]	3 [1-6]	124 [41-211]	129 [43-221]	2.1 [0.7-3.6]
South Dakota	1 [0-1]	0 [0-0]	7 [2-12]	8 [2-13]	0.8 [0.2-1.3]
Tennessee	5 [2-9]	5 [2-8]	173 [58-294]	183 [62-311]	2.1 [0.7-3.6]
Texas	79 [26-133]	99 [33-167]	650 [215-1,103]	828 [274-1,403]	2 [0.7-3.5]
Utah	1 [0-2]	0 [0-1]	98 [32-169]	99 [32-172]	2.1 [0.7-3.7]
Vermont	0 [0-0]	0 [0-0]	7 [2-12]	7 [2-12]	1.7 [0.5-3]
Virginia	5 [2-8]	2 [1-3]	242 [80-411]	249 [83-422]	2.7 [0.9-4.6]
Washington	0 [0-0]	0 [0-0]	197 [65-336]	197 [65-336]	2.7 [0.9-4.7]
West Virginia	3 [1-5]	1 [0-2]	41 [14-71]	45 [15-78]	2.1 [0.7-3.7]
Wisconsin	3 [1-5]	1 [0-2]	119 [40-203]	123 [41-210]	2.1 [0.7-3.5]
Wyoming	0 [0-0]	0 [0-0]	3 [1-5]	3 [1-5]	0.5 [0.2-0.8]
Contiguous US	276 [93-464]	351 [118-590]	9,720 [3,246-16,432]	10,347 [3,457-17,486]	2.8 [0.9-4.7]

ǂ State totals are rounded to nearest whole numbers. Contiguous US values are rounded after summing the unrounded state values.

* Uncertainty interval for health burden estimate. Calculation described in Methods Section 3.

‖Population Attributable Fraction (PAF) or fraction of total preterm birth incidences linked to $PM_{2.5}$ from the O&G lifecycle. Expressed as a percentage.

Supplementary Table 6. Cancer incidences from lifetime exposure to VOCs linked to major O&G lifecycle stages in 2017[‡].

State	Upstream + Midstream	Downstream	End-use	Lifecycle Total	Lifecycle PAF[||] (%)
Alabama	2	0	20	22	3.6
Arizona	1	0	27	28	4
Arkansas	2	0	9	11	2.8
California	22	13	263	298	9
Colorado	29	1	12	42	8.3
Connecticut	1	0	20	21	4.4
Delaware	0	0	5	5	4
District of Columbia	0	0	4	4	7.1
Florida	2	0	52	54	1.6
Georgia	2	0	71	73	6
Idaho	0	0	2	2	1
Illinois	6	1	34	41	2.6
Indiana	3	0	15	18	2.1
Iowa	1	0	4	5	1.1
Kansas	3	0	4	7	2.1
Kentucky	2	0	15	17	2.4
Louisiana	5	3	14	22	3.6
Maine	0	0	3	3	1.7
Maryland	2	0	33	35	6.2
Massachusetts	1	0	35	36	4.6
Michigan	4	0	27	31	2.4
Minnesota	2	0	18	20	2.7
Mississippi	2	0	12	14	3.4
Missouri	3	0	18	21	2.6
Montana	0	0	1	1	0.9
Nebraska	1	0	2	3	1.4
Nevada	0	0	12	12	4.4
New Hampshire	0	0	6	6	3.5
New Jersey	7	1	65	73	6.9
New Mexico	4	0	3	7	3.1
New York	14	1	117	132	5.3
North Carolina	2	0	49	51	3.7
North Dakota	4	0	0	4	5.8
Ohio	11	0	30	41	2.7

State	Upstream + Midstream	Downstream	End-use	Lifecycle Total	Lifecycle PAF‖ (%)
Oklahoma	9	1	11	21	4.4
Oregon	0	0	11	11	2.4
Pennsylvania	16	2	62	80	4.5
Rhode Island	0	0	5	5	3.9
South Carolina	1	0	22	23	3.4
South Dakota	1	0	0	1	0.9
Tennessee	3	0	31	34	3.6
Texas	70	11	88	169	6.5
Utah	1	0	9	10	4
Vermont	0	0	2	2	3.2
Virginia	3	0	42	45	5.3
Washington	0	0	29	29	3.8
West Virginia	3	0	6	9	2.7
Wisconsin	1	0	13	14	1.9
Wyoming	0	0	0	0	0
Contiguous US	249	36	1,334	1,619	4.3

ǂ State totals are rounded to nearest whole numbers. Contiguous US values are rounded after summing the unrounded state values.

‖Population Attributable Fraction (PAF) or fraction of total cancer incidences from CDC WONDER linked to HAPs from the O&G lifecycle.

Supplementary Table 7. NO_2-attributable pediatric (children aged 1-18 years) asthma incidences linked to major O&G lifecycle stages in 2017[‡,*].

State	Upstream + Midstream [UI]	Downstream [UI]	End-use [UI]	Lifecycle Total [UI]	Lifecycle PAF[‖] [UI]
Alabama	167 [31-247]	70 [13-104]	4,572 [835-6,793]	4,809 [879-7,144]	4 [0.7-5.9]
Arizona	8 [2-12]	0 [0-0]	7,047 [1,414-10,237]	7,055 [1,416-10,249]	13.7 [2.8-20]
Arkansas	54 [10-80]	5 [1-7]	581 [108-860]	640 [119-947]	3.8 [0.7-5.6]
California	149 [34-210]	361 [82-508]	40,464 [8,352-58,443]	40,974 [8,468-59,161]	16.4 [3.4-23.7]
Colorado	334 [70-480]	25 [5-35]	3,256 [644-4,747]	3,615 [719-5,262]	10.5 [2.1-15.3]
Connecticut	5 [1-8]	3 [1-4]	2,393 [437-3,555]	2,401 [439-3,567]	9.6 [1.8-14.3]
Delaware	5 [1-7]	12 [2-17]	646 [118-961]	663 [121-985]	10 [1.8-14.9]
District of Columbia	4 [1-5]	0 [0-1]	604 [113-892]	608 [114-898]	17.7 [3.3-26.2]
Florida	-2 [-4 to -1]	3 [1-5]	4,612 [831-6,872]	4,613 [828-6,876]	3.8 [0.7-5.7]
Georgia	13 [3-20]	7 [1-10]	7,600 [1,417-11,239]	7,620 [1,421-11,269]	10 [1.9-14.8]
Idaho	2 [0-3]	0 [0-0]	227 [40-341]	229 [40-344]	2.7 [0.5-4]
Illinois	83 [17-120]	81 [16-117]	8,536 [1,632-12,546]	8,700 [1,665-12,783]	10.9 [2.1-16]
Indiana	63 [12-93]	9 [2-14]	3,750 [696-5,549]	3,822 [710-5,656]	8.8 [1.6-13.1]
Iowa	5 [1-8]	1 [0-2]	685 [126-1,017]	691 [127-1,027]	4.9 [0.9-7.2]
Kansas	166 [32-244]	25 [5-37]	1,147 [211-1,702]	1,338 [248-1,983]	6.7 [1.2-9.9]
Kentucky	82 [16-120]	5 [1-8]	1,902 [352-2,817]	1,989 [369-2,945]	6.9 [1.3-10.3]
Louisiana	104 [20-154]	377 [70-558]	1,345 [245-2,000]	1,826 [335-2,712]	6.9 [1.3-10.3]
Maine	0 [0-0]	0 [0-0]	117 [49-159]	117 [49-159]	1.6 [0.7-2.2]
Maryland	47 [9-68]	8 [2-12]	5,498 [1,037-8,107]	5,553 [1,048-8,187]	13.1 [2.5-19.3]
Massachusetts	5 [1-7]	2 [0-3]	3,881 [1,656-5,178]	3,888 [1,657-5,188]	9.4 [4-12.6]
Michigan	49 [10-72]	5 [1-7]	5,165 [972-7,618]	5,219 [983-7,697]	8.2 [1.6-12.1]
Minnesota	8 [2-11]	3 [1-4]	2,737 [513-4,042]	2,748 [516-4,057]	8.9 [1.7-13.1]
Mississippi	10 [2-16]	13 [2-19]	331 [58-495]	354 [62-530]	2.3 [0.4-3.4]
Missouri	65 [13-94]	15 [3-21]	2,391 [450-3,526]	2,471 [466-3,641]	6.3 [1.2-9.3]
Montana	1 [0-2]	0 [0-0]	41 [7-61]	42 [7-63]	0.8 [0.1-1.3]
Nebraska	13 [3-19]	1 [0-1]	391 [77-570]	405 [80-590]	4.1 [0.8-6]
Nevada	0 [0-1]	0 [0-1]	2,586 [492-3,804]	2,586 [492-3,806]	12.8 [2.4-18.9]
New Hampshire	1 [0-1]	0 [0-0]	286 [120-385]	287 [120-386]	4.1 [1.7-5.5]
New Jersey	44 [10-63]	64 [14-91]	11,295 [2,265-16,418]	11,403 [2,289-16,572]	17 [3.4-24.7]
New Mexico	70 [14-102]	1 [0-1]	804 [164-1,164]	875 [178-1,267]	5.9 [1.2-8.5]

State	Upstream + Midstream [UI]	Downstream [UI]	End-use [UI]	Lifecycle Total [UI]	Lifecycle PAF‖ [UI]
New York	151 [33-215]	37 [8-52]	19,961 [4,029-28,976]	20,149 [4,070-29,243]	13.9 [2.8-20.2]
North Carolina	20 [4-30]	2 [0-3]	3,808 [708-5,633]	3,830 [712-5,666]	6 [1.1-8.9]
North Dakota	8 [2-12]	0 [0-0]	79 [15-117]	87 [17-129]	2.3 [0.4-3.3]
Ohio	167 [32-245]	30 [6-44]	6,386 [1,172-9,475]	6,583 [1,210-9,764]	9.2 [1.7-13.6]
Oklahoma	302 [57-446]	50 [9-73]	2,095 [383-3,112]	2,447 [449-3,631]	8.8 [1.6-13.1]
Oregon	0 [0-0]	0 [0-0]	1,566 [285-2,330]	1,566 [285-2,330]	7.8 [1.4-11.6]
Pennsylvania	491 [98-714]	132 [26-192]	9,438 [1,797-13,884]	10,061 [1,921-14,790]	12.1 [2.3-17.8]
Rhode Island	1 [0-1]	0 [0-1]	419 [176-563]	420 [176-565]	6.8 [2.8-9.1]
South Carolina	3 [1-4]	6 [1-8]	1,529 [272-2,285]	1,538 [274-2,297]	4.9 [0.9-7.4]
South Dakota	2 [0-2]	0 [0-0]	69 [12-104]	71 [12-106]	1.7 [0.3-2.5]
Tennessee	30 [6-44]	4 [1-6]	2,191 [399-3,257]	2,225 [406-3,307]	6.1 [1.1-9.1]
Texas	2,362 [471-3,435]	876 [175-1,274]	22,756 [4,283-33,577]	25,994 [4,929-38,286]	11.8 [2.2-17.4]
Utah	19 [4-27]	0 [0-0]	2,452 [481-3,583]	2,471 [485-3,610]	13.1 [2.6-19.2]
Vermont	0 [0-0]	0 [0-0]	23 [10-31]	23 [10-31]	0.7 [0.3-1]
Virginia	58 [11-86]	4 [1-6]	4,618 [841-6,867]	4,680 [853-6,959]	8.4 [1.5-12.4]
Washington	0 [0-0]	0 [0-0]	3,737 [710-5,503]	3,737 [710-5,503]	10 [1.9-14.7]
West Virginia	145 [28-213]	10 [2-14]	490 [94-720]	645 [124-947]	6 [1.1-8.8]
Wisconsin	5 [1-8]	4 [1-6]	1,891 [343-2,813]	1,900 [345-2,827]	6.4 [1.2-9.6]
Wyoming	10 [2-15]	2 [0-3]	48 [9-71]	60 [11-89]	2 [0.4-2.9]
Contiguous US	5,330 [1,098-7,693]	2,254 [464-3,253]	208,447 [40,473-305,357]	216,031 [42,035-316,303]	10 [1.9-14.6]

ǂ State totals are rounded to nearest whole numbers. Contiguous US values are rounded after summing the unrounded state values.

*Uncertainty interval for health burden estimate. Calculation described in Methods Section 3.

‖Population Attributable Fraction (PAF) or fraction of total pediatric (children aged 1-18 years) asthma incidences linked to NO_2 from the O&G lifecycle.

Supplementary Table 8. Absolute air pollutant exposure and attributable health burden linked to major O&G lifecycle stages by race and ethnicity in 2017.

Pollutant	Metric	Socio-demographic group	Upstream + Midstream	Downstream	End-use
$PM_{2.5}$	Exposure (μg m^{-3})	National Value	0.035	0.045	1.341
		Hispanic	0.042	0.064	1.603
		Asian	0.034	0.067	1.901
		Black	0.034	0.056	1.344
		Native American	0.036	0.027	0.821
		White	0.034	0.036	1.220
	Premature mortality (per 10 million people)	National Value	67.67	81.87	2,307.02
		Hispanic	59.55	85.53	2,073.01
		Asian	37.49	68.57	1,903.81
		Black	81.30	130.09	2,767.75
		Native American	85.50	49.41	1,590.97
		White	65.93	66.83	2,156.80
	Preterm birth incidences (per 100,000 births)	National Value	7.387	9.388	260.004
		Hispanic	8.333	11.765	290.474
		Asian	5.730	10.022	308.117
		Black	9.284	14.722	344.789
		Native American	8.617	5.657	178.821
		White	7.101	8.165	236.899
MDA8 O_3	Exposure (ppb)	National Value	0.233	0.026	2.438
		Hispanic	0.229	0.015	1.912
		Asian	0.169	0.007	1.834
		Black	0.234	0.047	3.013
		Native American	0.285	0.024	2.250
		White	0.239	0.028	2.534
	Premature mortality (per 10 million people)	National Value	12.90	1.63	133.64
		Hispanic	9.06	0.75	75.01
		Asian	6.73	0.54	73.19
		Black	11.32	2.49	142.15
		Native American	19.65	1.80	143.43
		White	14.54	1.86	153.44
NO_2	Exposure (ppb)	National Value	0.058	0.027	2.499
		Hispanic	0.078	0.038	3.176
		Asian	0.040	0.035	3.678
		Black	0.047	0.038	2.681

Pollutant	Metric	Socio-demographic group	Upstream + Midstream	Downstream	End-use
		Native American	0.082	0.016	1.583
		White	0.056	0.021	2.171
	Premature mortality (per 10 million people)	National Value	204.06	84.53	7,568.52
		Hispanic	195.91	88.23	7,618.32
		Asian	89.00	71.06	7,448.43
		Black	183.23	146.70	9,096.13
		Native American	303.30	55.47	4,565.28
		White	210.49	68.47	6,741.93
	Childhood asthma incidences (per 100,000 people)	National Value	6.97	2.95	273.77
		Hispanic	9.59	3.91	335.25
		Asian	4.68	3.60	399.89
		Black	5.85	4.32	307.10
		Native American	9.11	1.74	160.28
		White	6.60	2.35	238.33
VOCs	Exposure (μg m^{-3})	National Value	0.056	0.006	0.252
		Hispanic	0.072	0.011	0.299
		Asian	0.049	0.011	0.364
		Black	0.049	0.007	0.271
		Native American	0.066	0.004	0.157
		White	0.053	0.005	0.225
	Cancer incidences (per 10 million people)	National Value	7.82	1.14	41.99
		Hispanic	10.39	1.87	47.29
		Asian	6.99	1.91	56.59
		Black	6.79	1.34	47.21
		Native American	9.56	0.66	27.22
		White	7.36	0.83	38.29
